# *In Vitro* Infection of Pupae with Israeli Acute Paralysis Virus Suggests Disturbance of Transcriptional Homeostasis in Honey Bees (*Apis mellifera*)

**DOI:** 10.1371/journal.pone.0073429

**Published:** 2013-09-05

**Authors:** Humberto F. Boncristiani, Jay D. Evans, Yanping Chen, Jeff Pettis, Charles Murphy, Dawn L. Lopez, Michael Simone-Finstrom, Micheline Strand, David R. Tarpy, Olav Rueppell

**Affiliations:** 1 Department of Biology, University of North Carolina at Greensboro, Greensboro, North Carolina, United States of America; 2 Bee Research Laboratory, Agricultural Research Service of the United States Department of Agriculture, Beltsville, Maryland, United States of America; 3 Soybean Genomics and Improvement, Agricultural Research Service of the United States Department of Agriculture, Beltsville, Maryland, United States of America; 4 Department of Entomology, North Carolina State University, Raleigh, North Carolina, United States of America; 5 United States Army Research Office, Division of Life Sciences, Research Triangle Park, North Carolina, United States of America; University of British Columbia, Canada

## Abstract

The ongoing decline of honey bee health worldwide is a serious economic and ecological concern. One major contributor to the decline are pathogens, including several honey bee viruses. However, information is limited on the biology of bee viruses and molecular interactions with their hosts. An experimental protocol to test these systems was developed, using injections of Israeli Acute Paralysis Virus (IAPV) into honey bee pupae reared ex-situ under laboratory conditions. The infected pupae developed pronounced but variable patterns of disease. Symptoms varied from complete cessation of development with no visual evidence of disease to rapid darkening of a part or the entire body. Considerable differences in IAPV titer dynamics were observed, suggesting significant variation in resistance to IAPV among and possibly within honey bee colonies. Thus, selective breeding for virus resistance should be possible. Gene expression analyses of three separate experiments suggest IAPV disruption of transcriptional homeostasis of several fundamental cellular functions, including an up-regulation of the ribosomal biogenesis pathway. These results provide first insights into the mechanisms of IAPV pathogenicity. They mirror a transcriptional survey of honey bees afflicted with Colony Collapse Disorder and thus support the hypothesis that viruses play a critical role in declining honey bee health.

## Introduction

Over the last years, the declining health of the honey bee (Apis mellifera) and other pollinators has caused concern all over the world. Particularly over the last six years, honey bee health has shown alarming rates of deterioration [1], [2], [3], questioning the sustainability of our food production system. There are many possible threats to honey bees health, including pesticides, malnutrition, management stress, and pathogens [3], [4], [5], [6], [7].

Numerous studies suggest that novel or emerging pathogens play a role in honey bee health declines [1], [2], [3], [7], [8], [9]. However, insufficient knowledge on honey bee pathogens compromises our ability to assess their importance and to develop control measures. This is particularly true for honey bee viruses although their importance for honey bee losses has become evident in recent years [6], [7], [8], [10], [11], [12]. Specifically in combination with the ectoparasitic bee mite Varroa destructor [13], [14], [15] that serves as a vector, several viruses appear to become more virulent [16], [17], [18]. Viruses may cause covert infections [19] and were considered mostly harmless until Varroa mites were introduced to A. mellifera populations almost 30 years ago [20], [21], [22]. The increased virulence of viruses has been confirmed experimentally by direct inoculation of bees with viruses [23], [24], [25], [26], [27], opening an important research field to explore.

Approximately twenty honey bee viruses have been described so far [4], [16], [28], [29], affecting the morphology, physiology, and behavior of bees. Most belong to the families Dicistroviridae [30] and Iflaviridae in the order Picornavirales. Viruses in these families have a positive sense RNA genome, covered by an icosahedral, pseudo T = 3 structure symmetry capsid (around 30 nm) that is responsible for RNA protection, host specificity, and tissue infection. Picornaviruses are well known for their capacity to shut off the translational system of their host cells, by cleavage of translation factors leading to a decrease in cap-dependent host translation, a conserved replication strategy among all members studied to date [31], [32]. Picornavirus infection also triggers host-immune responses (i.e., PKR) that result in decreased cap-dependent (host) translation. Picornaviruses circumvent this immune response by encoding an internal ribosomal entry site (IRES) which is recognized and translated by the host machinery (non-canonical translation) [30], [32]. Over time, the accumulation of produced virus particles and repression of the synthesis of essential cell components lead to cell death in most cases [32].

Little is known about the specific biology of the viruses in these families that infect honey bees, although they contain important bee pathogens, such as Deformed Wing Virus (DWV) and Israeli Acute Paralysis Virus (IAPV). IAPV has previously been associated with the unusual honey bee disappearance syndrome called Colony Collapse Disorder (CCD) [8] and is frequently seen in many honey bee pathogen surveys [6], [7]. Despite the importance of IAPV and the feasibility to work with IAPV in the laboratory [25], [33], little is known about IAPV’s interactions with its host and resulting pathogenesis.

In general, the lack of adequate tools for honey bee virus research has hampered our understanding of basic biology of the relevant viruses and little is known about the molecular bases of honey bee viruses replication and pathogenesis [18], [28]. Therefore, many assumptions regarding their replication are made based on other picornaviruses (e.g., cricket paralysis virus [34], [35] and human poliovirus [32]), highlighting the need of specific, mechanistic studies on honey bee viruses [36]. Focusing on IAPV, we report here the development of an inoculation method of in-vitro reared honey bee worker pupae that provides the basis for mechanistic, in-depth studies of honey bee viruses. We report acute but variable disease symptoms, compare viral replication among pupae of two colonies and patrilines within these colonies, and report on measures of gene expression in response to viral infection that indicate major disruption of cellular homeostasis.

## Materials and Methods

### Virus Preparation, Quantification and Electron Microscopy

Initially, approximately 20 adult bees from a heavily IAPV-infected colony were frozen in liquid nitrogen, ground to a fine powder, and homogenized in 10 ml extraction buffer (0.1 M potassium phosphate buffer, pH 7.5, 0.2% diethyldithiocarbamate, 1/5 volume of diethyl ether). Emulsification ensued by adding 5 ml carbon tetrachloride and centrifuging at 10,000 rpm for at 4°C for 10 minutes (Rotor: Sorvall RC-5B) and collecting the supernatant.

The supernatant containing viruses was filtered through a 0.2-micron filter (milex-GS, Millipore, #SLGS033SS) to remove small tissue debris, fungi, and bacteria. The filtrate was then centrifuged at 30,000 rpm (61,740 RCF) in a Beckman LB-70M ultracentrifuge with a 70.1/Ti rotor for six hours at 4°C to pellet picornavirus particles. The pellet was resuspended in 0.2 ml of PBS and centrifuged against a CsCl gradient (0.44 g/ml) at 52,000 rpm (185,000 RFC) overnight (Beckman LB-70M ultracentrifuge, 70.1/Ti rotor). The fractions containing virus particles were dialyzed using “Slide-A-Lyzer Dialysis Cassettes” against cold (10°C) 0.2 ml of PBS overnight. RT-PCR was conducted to test for the presence of Acute Bee Paralysis Virus (ABPV), Israel Acute Paralysis Virus (IAPV), Kashmir Bee virus (KBV), Sacbrood virus (SBV), Chronic Bee Paralysis Virus (CBPV), Black Queen Cell Virus (BQCV), and Deformed Wing Virus (DWV). IAPV, and small amounts of ABPV, DWV, and BQCV were detected in the purified viral solution (positive amplification with PCR primers). Viral quantification was performed by absolute quantification using the Standard Curve Method as described previously [37] [38]. 5.0 µl of viral solution was examined for the presence of virus particles and their phenomenological characterization by electron microscopy. Virus particles were negatively stained with 2% Uranyl Acetate on a formvar-coated Ni grid and viewed in a Hitachi H-7000 electron microscope at 150,000X to 200,000X.

**Figure 1:**
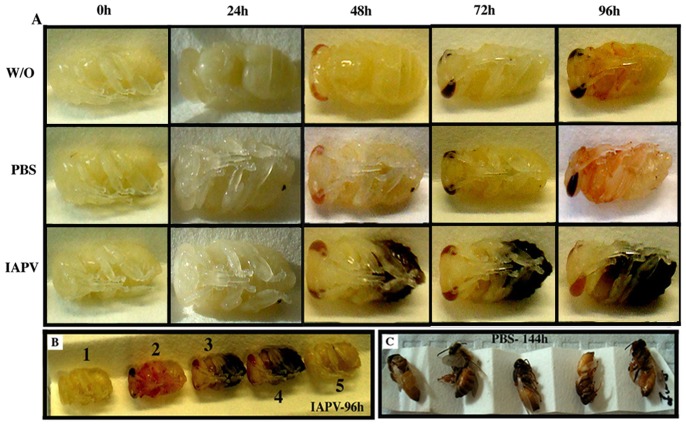
Experimental study system to follow virus pathogenicity during honey bee pupal development in the laboratory. [A] Development of IAPV-inoculated (IAPV), PBS-injected (PBS), and negative control (W/O) individuals. The IAPV group shows progressive symptoms of disease, compared to the normally developing PBS and control group. [B] Close-up of the variable symptoms of IAPV replication in white eye honey bee pupae: Complete cessation of development with no visual evidence of disease (1), Apparently normal development (2), Rapid darkening of different body parts (3,4), Darkening and hindered development combined (5). [C] Control bees are completing metamorphosis.

IAPV replicates readily in pupae [25]. Therefore, white-eyed pupae were inoculated for virus propagation, using 1.0 µl of the viral suspension per pupa. After 4 days of incubation, with disease symptoms apparent (Figure 1), viruses were purified using the approach outlined above. qRT-PCR showed ∼10^5^ more IAPV genomes than the second most detected virus, DWV after a single round of virus injection pupal amplification, and isolation. This procedure was repeated twice using pupae from very strong hives to further reduce contaminating viruses and increase the amounts of IAPV. The high concentration of IAPV over other honey bee viruses in these purifications allowed us to strongly dilute the inoculum, decreasing the chances of cross inoculation with another virus. In the experiments described below, we injected 10^4^ IAPV genome equivalents keeping the probability of cross contamination at negligible levels.

### RNA Extraction and qPCR Parameters

Pupae were individually homogenized and submitted to total RNA extraction, using TRIzol® (Invitrogen, Carlsbad CA) following the manufacturer’s protocol. The resultant RNA pellets were resuspended in diethyl pyrocarbonate-treated water in the presence of RNase inhibitor (Invitrogen, Carlsbad CA) and treated with DNase I (Invitrogen, Carlsbad CA) to remove any contaminating DNA. The resulting RNA was quantified on a Nanodrop spectrophotometer (Thermo Scientific, Wilmington, DE). First-strand cDNA was then synthesized by incubating 2µg of total RNA per sample in a 96-well plate with master mix containing 50 U Superscript II (Invitrogen, Carlsbad, CA), 2 nmol dNTP mix, 2 nmol poly(dT)18, and 0.1 nmol poly (dT) (12–18) at 42°C for 50 minutes followed by 15 minutes at 70°C as described previously [39]. The cDNA was diluted 1∶5 with molecular-grade water.

The primers used in this study were validated for relative quantification of the target genes and are commonly used in honey bees [39], [40], [41]. Reactions to amplify the cDNA products were conducted in 96-well plates using the Applied Biosystems Step One Real Time PCR machine (Applied Biosystems, Carlsbad, California). One microliter of diluted cDNA from each sample was used as a template for RT-qPCR reactions using SYBR Green™ (Applied Biosystems, Carlsbad, California) following the manufacturer’s protocols. The reactions were conducted under a fixed thermal protocol consisting of 3 minutes at 95°C, followed by 40 cycles of a three-step protocol of 95°C for 20 sec, 60°C for 30 sec, 72°C for 1 minute. Fluorescence measurements were taken at each cycle during the last 72°C step. This procedure was followed by a melt-curve dissociation analysis to confirm the specificity of the reactions.

### Experimental Design

Based on the results of the preliminary experiments 1 and 2 (Preliminary Experiments S1), a more extensive IAPV inoculation experiment was designed to study the time line of infection and associated gene expression patterns, and to assess bees for variability in IAPV susceptibility.

**Figure 2:**
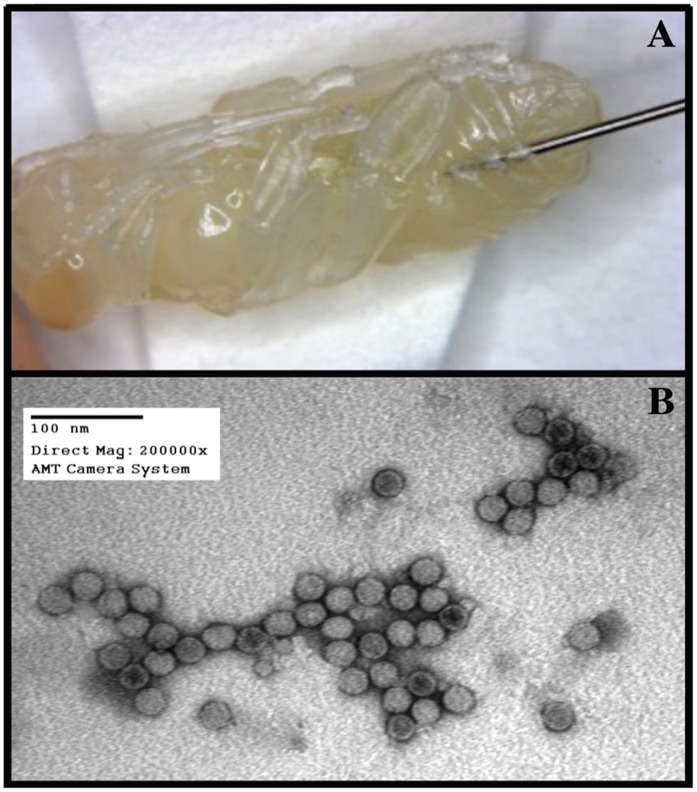
To enrich for IAPV from honey bees of typically mixed infections, repeated cycles of pupal inoculations and later virus purification from the inoculated pupae were performed. The preferential replication of IAPV during this procedure resulted in virus purifications with negligible levels of contamination from other viruses. [A] Inoculation of honey bee pupae with IAPV. Even though the injection apparatus varied among experiments (see main text), the basic injection site and methodology shown were identical. [B] Electron microscope image of purified IAPV sample, showing clean and uniform virus particles (full and empty particles) around 27 nm.

One microliter of the inoculant (PBS as control or virus solution containing 10^4^ genome equivalents of IAPV) was injected using a NanoJet™ syringe pump (Chemix, USA) with an infusion flow rate of 0.1µl/sec, following manufacturer’s parameters. The needle was inserted in the lateral abdomen between the fourth and fifth tergite of young, white-eye honey bee pupae (Figure 2A).

Two strong, IAPV-free hives were selected from the UNCG research apiary, representing two distinct sources of bees for the experiment. From each hive, 200 white-eye pupae were collected for each of the following treatment groups: without inoculation (W/O), PBS inoculated (PBS), and IAPV inoculated (IAPV). From each treatment group and hive, 50 bees were frozen at 0 h, 5 h, 20 h and 48 h after inoculation and a subset of these samples was individually analyzed for viral titers and gene expression patterns. The first time point directly after inoculation was used as a control of the initial states of the bees in the experimental and control groups. The time point of five hours post-infection was chosen to measure the virus impact before completion of the replication cycle, based on the assumption that IAPV follows the picornavirus family average timing for a replication cycle, of 7–12 hours [32], [34], [35]. Any gene expression changes at this time point represent the bees’ response to inoculation without complications from virus-related tissue damage. The time point of 20 h post-infection was considered representative of events after one complete cycle of virus replication, and the 48 h time point represents the established diseased state, characterized by visual symptoms.

Based on the results of the preliminary experiments (Preliminary Experiments S1), we tested the effect of IAPV injection on gene expression of six commonly used reference genes that have been reported to be constantly expressed across different experimental conditions [4], [7], [41]. We studied the transcription of Actin, ribosomal 28S RNA, ribosomal 18S RNA, ribosomal protein RPS5, MGST1, and Histone H2A, under IAPV infection. Histone H2A is not common used in honey bees, but it was added to our experiment because its expression is constitutive and cell-cycle independent, and it is commonly used on other models [42]. The sequences of utilized H2A primers are: 5′-AAAGGAAATTACGCAGAACGA-3′ (H2A Forward) and 5′-CGGCTAAATATTCCATAACGG-3′ (H2A Reverse). In addition, the titers of IAPV and DWV were quantified in these samples.

### Patrilines Genotyping

One hind leg was removed from each pupa and stored at -20°C before DNA extraction to determine the subfamily (patriline) for each individual. DNA was extracted from each leg using a standard Chelex 100® method [43]. Briefly, each sample was incubated for 60 min at 55°C, 15 min at 99°C, 1 min at 37°C, and 15 min at 99°C in 150 µl of a 5% Chelex 100® solution with 5 µl 0.35 mg/µl proteinase K.

Subfamily identification for each sample was determined using microsatellite alleles following previously described methods [44]. This genomic paternity analysis was conducted using two multiplex PCR reactions (Plex 1 and Plex 2) with 10 µl reaction volumes containing approximately 100 ng sample DNA, 1×PCR buffer (Takara® without MgCl_2_), 1 mg/ml BSA, 1.5 U Taq polymerase, 300 µM dNTPs, and either 1.5 mM (Plex 1) or 1.1 mM (Plex 2) MgCl_2_. Following [45], primer sets in Plex 1 included 2.0–2.5 pMol Am061, Am052, Am010, and Am553, and primer sets in Plex 2 included 2.5–3.5 pm Am043, Am098, Am125, and Am059. All reactions were performed using a Thermo® Px2 thermocycler with 7 min at 95°C followed by 30 cycles of 30 sec at 95°C, 30 sec at 55°C (Plex 1) or 54°C (Plex 2), and 30 sec at 72°C, then a final 10 min at 72°C. The PCR products were run on an ABI 3730® DNA Analyzer at the Genomic Sciences Laboratory at NCSU. Data was acquired with Genemapper 4.0 (ABI) to score microsatellite fragment sizes. Loci with poor amplification were excluded from analyses and only samples for which more than half of the loci could be scored were used for analysis. The data were analyzed with the computer package Colony 1.2 to assign subfamily membership to each sample [46].

### Gene Expression Analysis

All experiments revealed that the absolute quantities (Ct values) of the standard reference genes were affected by the IAPV treatment (see Results) and that they did not fulfill the criterion of expression stability. Therefore, a larger set of potential reference genes was evaluated in the main experiment (Raw Data S1). However in the absence of an internal control, the transcript level of these genes and IAPV could not be normalized by the customary ΔCt or ΔΔCt methods [47], [48]. Instead, transcripts were evaluated by Ct values, based on the assumption that the amount of template after quantification and appropriate dilution did not differ systematically among treatment groups. To ensure consistency, a fixed fluorescence threshold for each gene and experiment was determined manually to avoid inter RT-qPCR runs inconsistencies. Tests of technical error indicated a high replicability for several genes, with variation between replicate Ct values of 1.3% on average (minimum: 0.002%, maximum: 2.6%).

All statistical analyses of this study were done using The R Stats Package, version 2.15.0, http://www.r-project.org/ or with SPSS 20.0 (IBM). Heat maps were generated by Heatmap.Plus R Package version 1.3. Patterns of gene expression were analyzed with parametric linear models, using time and treatment as fixed effects. Bonferroni and Scheffe’s post-hoc tests were performed and did not differ in their results. In the experiment, the virus titers of inoculated individuals were compared among colonies and patrilines within colonies. Patrilines that were represented by only one individual were omitted from the patrline but not the colony analysis. Thus, separate ANOVAs were used instead of one nested ANOVA.

## Results

### IAPV Purification

Attempts to isolate pure IAPV directly from naturally infected adult bees were unsuccessful due to co-infection of the bees with other honey bee viruses. PCR tests resulted repeatedly in positive amplification of multiple viruses, such as BQCV, ABPV, and DWV. Co-infection seems to be the rule rather than the exception and it is generally rare to find bees infected with a single virus [49]. However, our artificial inoculation of pupae led to selective increases of IAPV, relative to the other viruses. After three rounds of pupae inoculation and subsequent virus purification, the amount of IAPV was at least 10^5^ genome copies higher than all other common honey bee viruses found in our initial inoculum (BQCV, ABPV, DWV). After serial dilutions, IAPV was the only virus that could be detected by PCR. Electron Microscopy analysis of this sample showed uniform viral particles around 27 nm (Figure 2B), consistent with picornavirus particles. Sequence analysis verified these particles to be IAPV.

### *In vitro* IAPV Infection System Standardization

The site for injection of the virus inoculum into the honey bee was chosen based on the ability of the pupae to complete development to become an adult after sham injections. When the junction between the last abdominal sternites (Figure 2A) was selected more than 95% of bees were able to complete development after PBS inoculation. This region is very soft, enabling smooth penetration of the needle with little physical damage to the pupae. In addition, Varroa destructor nymphs were often observed in this same region when pupae were prepared for inoculations, suggesting that this area is an attractive feeding site.

**Figure 3:**
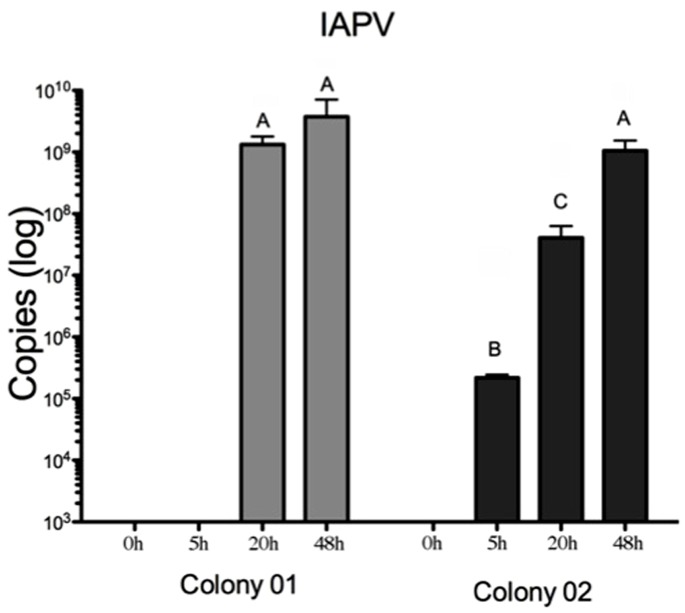
IAPV titer increases in IAPV-inoculated bees from the two studied colonies suggest that the colony source influences the IAPV replication kinetic. From initially undetectable levels, IAPV increases more gradually in the second colony, resulting in significantly lower titers 20 hours after inoculation than in the first colony. Each bar represents an experimental group of individually assessed bees. The bars with different letters are significantly different (ANOVA post-hoc tests, p<0.001).

**Figure 4:**
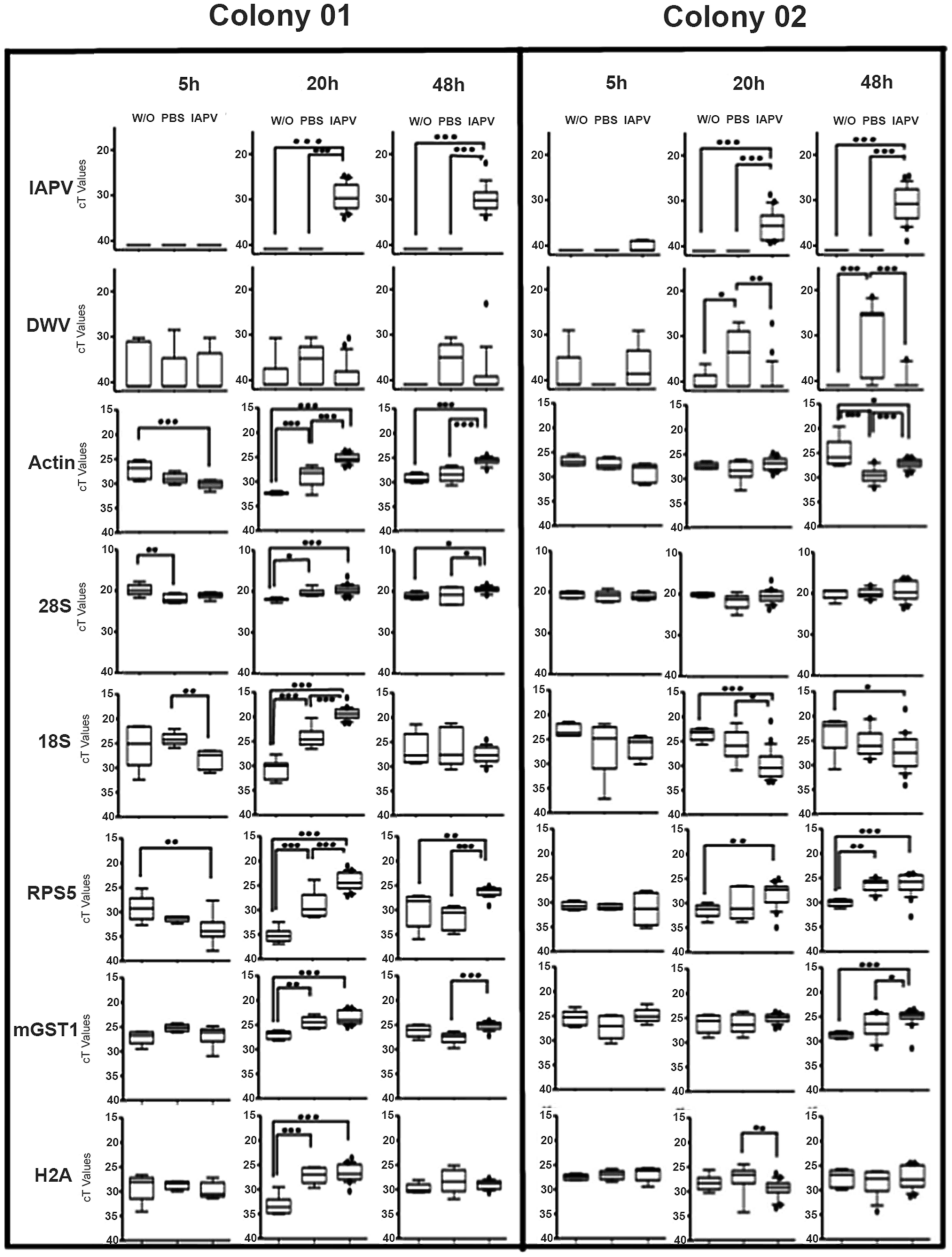
Transcript levels showed colony, time, and treatment effects. The observed expression patterns indicate that IAPV infection disturbs ribosomal biogenesis and other cellular functions. IAPV-injected bees of colony 01 suffered a fast build-up of IAPV and showed an almost ubiquitous up-regulation of genes. The overall pattern is more complicated in colony 02, possibly due to a more gradual IAPV build-up and confounding effects of DWV infection. The median bar shows the median value, and the boxes are the 25–75 percentiles; error bars are the 10–90 percentiles, and outliers are indicated as dots. Significance of post-hoc comparisons are indicated by * (<0.05), ** (<0.01), or *** (<0.001).

In the standardization process both controls, without inoculation (W/O) and PBS buffer injected bees (PBS), developed normally (Figure 1A), culminating in full development after 144 hours (Figure 1C). IAPV-inoculated bees showed strong but variable symptomatology over time (Figures 1A and 1B), inhibited metamorphosis, and ultimately death. Symptoms ranged from a complete cessation of development with no visual evidence of disease (Figure 1B-1), rapid darkening of body parts (Figure 1B–3 and 1B–4), simultaneous darkening and hindered development (Figure 1B–5), to apparently normal development (Figure 1B–2) with eventual sudden death. IAPV titer increased in all inoculated bees but no correlation between symptomatology and virus titer determined by RT-qPCR at the end of the experiment was observed.

### Variation in IAPV Susceptibility

The experiment investigated IAPV infections in pupae from two unrelated colonies to compare these two colonies and patrilines within the colonies. RT-qPCR analyses showed no initial evidence of IAPV infection in either experimental colony and even the initial inoculum was below our detection limit (Figure 3). A two-factorial ANOVA indicated that the two colonies differed significantly in the build-up of virus titers (F^Colony^_(1,104)_ = 5.3, P = 0.023; F^Time^_(3,104)_ = 69.7, P<0.001; F^Interaction^_(3,104)_ = 8.0, P<0.001). Specifically, a significant difference between the colonies was found at 20 hours (F^Colony^_(1,42)_ = 39.2, P<0.001; Figure 3). Post-hoc tests also revealed a significant difference among all time points, except between 0 and 5 hours. Within colonies, some patriline differences were suggestive (Figure S1) but not significant after Bonferroni correction (Colony 1: F^20 h^_(2,12)_ = 4.5, p_uncorrected_ = 0.025; F^48 h^_(3,14)_ = 0.2, p_uncorrected_ = 0.917; Colony 2: F^20 h^_(1,12)_ = 0.5, p_uncorrected_ = 0.476; F^48 h^_(5,18)_ = 2.2, p_uncorrected_ = 0.094). DWV was detected in 55 samples and its titers were only influenced significantly by treatment (F^treatment^_(2,181)_ = 4.5, p = 0.012, with an interaction between treatment and time (F^treat×time^_(6,181)_ = 4.5, p<0.001): Treatment only affected DWV titers after 20 and 48 hours and post-hoc tests showed that the PBS-injected individuals had significantly higher DWV titers than the W/O and the IAPV-inoculated bees (Figure 4).

Symptomatic differences were also observed between the two colonies (Table 1). Generally, pupae from Colony 1 showed some evidence of developmental completion, as evidenced by the presence of brown eye pigmentation and darkened abdomens. Pupae from Colony 2 showed higher development debilitation: few individuals developed eye pigmentation or darkened abdomens. No correlations between virus titer and symptomatology were found.

**Table 1:** IAPV symptomology between two different hives after 48 hours of infection.

	Darkening	Hindered development	Hindered development and darkening
Colony 01	27/50	7/50	3/50
Colony 02	3/50	43/50	0/50

### Transcriptional Consequences of IAPV Infection

Three-factorial ANOVA revealed a significant treatment effect on the expression on all six genes (Table 2). In general, gene expression also differed among time points but the differences for Actin were not significant. In contrast, the two colonies only differed in Actin expression (Table 2). Post-hoc tests of main treatment effects showed significantly higher gene expression in the IAPV-inoculated bees compared to the two control groups for Actin, 28S rRNA, and mGST1. Conversely, 18S rRNA was significantly less expressed in IAPV-inoculated bees than in both control groups. For Histone H2A, significantly lower expression was found in the untreated group than in the PBS and virus injected group, and all treatment groups differed significantly in the RPS5 expression in the following order: “control group”<“PBS-injected”<“IAPV-injected”. Post-hoc test results for time effects were more complex (Results S1).

**Table 2:** Main effects* on the expression of six common reference genes.

Gene	Time Effect	Treatment Effect	Colony Effect	Correlation with IAPV titer
Actin	F_(3,181)_ = 2.5, P = 0.058	**F_(2,181)_ = 8.5, P<0.001**	**F_(1,181)_ = 7.5, P<0.001**	**η_p_^2^ = 0.09, p<0.001**
28S rRNA	**F_(3,181)_ = 3.0, P = 0.032**	**F_(2,181)_ = 5.7, P = 0.004**	F_(1,181)_ = 0.07, P = 0.787	**η_p_^2^ = 0.16, p<0.001**
18S rRNA	**F_(3,181)_ = 9.1, P<0.001**	**F_(2,181)_ = 9.2, P<0.001**	F_(1,181)_ = 3.5, P = 0.062	**η_p_^2^ = 0.11, p<0.001**
RPS5	**F_(3,181)_ = 14.6, P<0.001**	**F_(2,181)_ = 16.0, P<0.001**	F_(6,181)_ = 1.4, P = 0.243	**η_p_^2^ = 0.17, p<0.001**
mGST1	**F_(3,181)_ = 10.1, P<0.001**	**F_(2,181)_ = 9.2, P<0.001**	F_(1,181)_ = 0.02, P = 0.876	**η_p_^2^ = 0.06, p = 0.001**
Histone H2A	**F_(3,181)_ = 3.5, P = 0.016**	**F_(2,181)_ = 11.1, P<0.001**	F_(1,181)_ = 3.3, P = 0.069	**η_p_^2^ = 0.18, p<0.001**

* Significant effects in bold.

The ANOVA models also revealed many significant interaction terms (Results S1), indicating time-specific and colony-specific treatment effects (Figure 4). The entire ANOVA model explained most of the gene expression variation for RPS5 (65.8%), followed by Actin (62.1%), mGST (40.7%), H2A (38.0%), 18S rRNA (35.0%), and 28S rRNA (18.9%). The additionally performed ANCOVAs revealed that all associations between IAPV and transcripts were also significant, independently of treatment or timing (Table 2). No correlations between gene expression and symptomatology were found.

## Discussion

Honey bee viruses play an important role in the recent declines in honey bee health [8], [9], [16], [17], [50], [51] but very little is known about how virus infections damage honey bees. The developed study model is a crucial step to much-needed mechanistic studies of honey bee viruses. On the one hand, honey bee pupae do not require feeding and can be easily maintained under laboratory conditions until full development into adult. They are highly relevant in the pathology of several viruses [16]. On the other hand, IAPV has proven an excellent choice because its preferential replication in pupae [25] enables the production of inoculum that is virtually free of contaminating viruses. In addition, IAPV is relevant for bee health [8] but it is not ubiquitous in the bee population, which makes it possible to set up experiments with IAPV-free bees.

Our experiment showed significant differences in IAPV replication between the two studied colonies, and also suggested patrilineal variation, although small sample sizes per patriline precluded significance after correction for multiple testing. Environmental factors are not to be disregarded and can include colony propolis [52] and pesticide [53] levels, or larval nutrition [54]. Colony 01 showed a more abrupt increase in virus titers, while IAPV increased more gradually in colony 02. However, the relative resistance of bees from colony 02 only delayed IAPV build-up and the IAPV titers were invariably high in pupae after 48 hours. The identification of genotypic variation in virus susceptibility would improve the prospect for selective breeding to improve honey bee health. In any case, our study demonstrates significant heterogeneity in virus amplification and gene responses (see below), highlighting the importance of standardization in honey bee health studies.

Distinct symptomology patterns were observed between the two colonies (Table 01). It is not clear whether virus-induced tissue damage and necrosis or melanization as part of the immune response is responsible for the observed darkening. Melanization is key component of insect immune response and is active in antiviral immunity [55], [56], [57]. Melanization would be predicted to correlate with resistance to IAPV, contrary to our observations of the darkening of larvae. Therefore, necrosis or other forms of cell death are a more likely explanation for the tissue darkening [58].

Quantifying gene expression responses to IAPV infection in the honey bee pupa according to standard protocols was complicated because IAPV infection significantly affected all investigated reference genes in all experiments (Preliminary Experiments S1 and Figure 4), precluding their meaningful incorporation into ΔCt or ΔΔCt analyses [48]. Even though relative quantification has considerable advantages and is used almost universally, it depends on appropriate references [59], which were not available for our study. Therefore, we relied on absolute quantification after standardizing the amount of RNA in each experiment. The Ct values were converted to another measure of absolute quantification (copy number) for IAPV by comparison to a standard curve. Variation in cDNA synthesis or other inequalities among samples might have contributed some experimental error. However, it is highly unlikely that technical errors are responsible for the observed significant differences among our experimental treatments, particularly given the consistency among our three separate experiments. Thus, we conclude that Ct values are most appropriate for our study and absolute quantification is necessary in studies of the transcriptional response to virus infections in honey bees. Caution needs to be exerted in general when interpreting relative gene expression patterns with respect to virus infections in honey bees and other organisms [60], [61].

The investigation of multiple reference genes confirmed the earlier conclusions that basic cellular pathways were significantly being affected by IAPV infection (Preliminary Experiments S1). Interestingly, the transcription of many genes in the PBS-injected bees was intermediate between the negative control and the IAPV group, demonstrating an effect of wounding itself. Overall, the expression of all genes was affected by time, although for Actin this effect was non-significant in the full factorial model. In contrast, Actin was the only gene that exhibited an overall expression difference between the two colonies. Furthermore, all genes were significantly associated with IAPV titers, independently of the treatment effects. In sum, all analyzed genes failed to fulfill the criteria for a reliable reference gene and instead indicated a profound disruption of fundamental cellular processes by IAPV. In addition to treatment effects, the expression of the putative reference genes also changed over time or was dependent on genotype. This transcriptional instability of putative reference genes might present a general disadvantage of the pupa as study system because the ongoing metamorphosis presumably affects numerous genes, independently of any treatment effects [62].

The biological interpretation of the main effects of host colony, time, and treatment is complicated by numerous significant interaction effects observed. For Actin all interactions among the three factors were significant and for the 28S rRNA no significant interactions were observed. The other four genes all showed a significant 3-way interaction and one or two 2-way interactions. Interactions between time and treatment are not surprising for any transcript because the treatment effects only appear at the later stages of the experiment. However, interactions between colony and treatment confirm the finding that source colony significantly affects the interaction between IAPV and its host. Bees of the more resistant colony 02 showed a down-regulation of the 18S rRNA by IAPV injection. In contrast, the transcript was increased by IAPV injection in bees from the more susceptible colony 01. Similarly, for most other transcripts, the strongest up-regulation by IAPV occurred after 20 hours in colony 01 but after 48 hours in colony 02. Further experiments are needed to determine the causal relationships among host genotype and environment, gene expression patterns, and IAPV replication.

The observed gene expression patterns could be due to viral manipulation of the cells to increase virus replication or present cell compensatory responses to IAPV infection. Typically, picornaviruses express a protease that cleaves the scaffold eIF4G initiator factor. This process inhibits the 5′ cap mediated translation of cellular peptides and redirects the cell translational machinery to viral mRNAs that depend on Internal Ribosomal Entry Sites (IRES)-mediated translation [30], [31]. The protease-mediated shut-down of cellular translation is widespread [63], [64] and homologs of the protease gene have been identified in all members of the dicistroviruses so far [30]. However, direct evidence for a translational inhibition that increases transcriptional activities via feedback loops is so far missing for all honey bee viruses and insect picornaviruses in general. RPS5 is a key component for IRES recognition in the dicistrovirus family [65], [66], [67]. The up-regulation of this gene benefits virus replication directly, suggesting that RPS5’s strong and consistent up-regulation may be directly induced by IAPV. However, the widespread transcriptional response to IAPV also suggests that the cell may respond to the lack of certain cell components by increasing their transcription. The up-regulation of Actin, MGST1, and the histone H2A in most experimental groups suggests a far-reaching, although variable, response in a range of basic cellular processes in addition to a disturbance of the ribosomal biogenesis pathway discussed below. More research is needed to understand these processes and it variability among environments and genotypes.

The three components of the ribosomal biogenesis pathway studied exhibited different responses to IAPV injection. While 28S rRNA, and RPS5 transcripts were invariably increased after IAPV replication (20 and 48 hours post-injection), 18S rRNA transcripts were decreased in colony 02 at both time points and were only increased in the more susceptible colony 01 at the first time point. The reason for these disparities is unclear, particularly because the 18S rRNA and 28S rRNA transcripts are derived from a polycistronic precursor mRNA [68]. However, the regulated balance between small and large ribosomal subunits [69] is controlled by independent maturation pathways [70] and IAPV presumably affects these pathways differently. The differences between colonies may indicate that the more resistant individuals may have either resisted transcriptional manipulation by IAPV or dedicated more cellular resources to immediate immune functions instead of ribosomal biogenesis. Consistent with this interpretation, gene expression patterns converged between the two colonies at the later time point.

Ribosome biogenesis is a highly complex and energetically costly pathway that is essential for all eukaryotic cells [70]. It is highly regulated and integrated with other cell functions, such as p53 signaling, and deregulation of ribosomal biosynthesis has been associated with oncogenesis [71] and apoptosis [72]. Apoptosis is a widespread cellular response to virus infection [73] and could explain some of the observed differences in IAPV symptomology. On the other hand, viruses can also directly interfere with the ribosomal biogenesis pathway by either up- or down-regulation [74], [75]. In any case, our result of a disturbance of the ribosomal biogenesis corroborates an important microarray survey of transcripts in the honey bee intestine that has linked picornaviruses and 28S rRNA transcript abundance to Colony Collapse Disorder [9].

The injection of PBS served as an experimental control to account for the effect of wounding during IAPV inoculation. For all genes, the observed transcription patterns of the PBS-injected bees were intermediate between the IAPV-injected and the negative control group. This observation may suggest that a similar disruption of basic cellular functions occurs in response to wounding and cellular trauma, resulting in profound changes at the transcriptome level [76]. However, our data show also an increase of DWV titers in the PBS-injected pupae over time and relative to both other treatment groups. Increased DWV titers in response to wounding have been observed before [77]. The increase of another picorna-like virus may have triggered responses in the PBS-injected bees that were similar to the IAPV injection, supporting a similar gene expression pattern observed between PBS group and IAPV injected bees compared to the negative control groups. In contrast to the PBS-injected bees, the IAPV-injected bees did not show an increase in DWV titers, suggesting that IAPV or cellular responses to IAPV interfere with DWV replication [78].

In summary, this study introduces an important model system to advance mechanistic studies on virus-host interactions in insects. It is particularly valuable to study honey bee viruses and their role in compromising honey bee health. The results demonstrate significant variability and indicate sources for this variability. The transcriptional analyses show profound, correlated perturbations of basic cellular functions and call into question the use of typical reference genes in this system. The investigated responses to IAPV inoculation in honey bees seem typical for picornavirus infections and provide a first step towards understanding the basic biology of this important honey bee virus. More detailed studies need to follow to manipulate virus and host and assess host responses to IAPV at the systemic level.

## Supporting Information

Figure S1 **Patriline differences of IAPV titers within colonies suggest a genetic basis for virus resistance in honey bees.**
(JPG)

Preliminary Experiments S1 **Two experimental inoculations of a limited number of bees with IAPV suggested that viral infection causes broad-scale alterations of transcript patterns, including immune and reference genes.**
(PDF)

Raw Data S1 **Raw Ct values for all qPCRs run in the main experiment.**
(PDF)

Results S1 **The expression of genes evaluated by full, 3-factorial ANOVAs indicated that developmental time is a significant factor, that was as important as treatment but for most genes interacted with treatment.**
(PDF)
