# Supplementary figures and images for: In Vitro Infection of Pupae with Israeli Acute Paralysis Virus Suggests Disturbance of Transcriptional Homeostasis in Honey Bees (Apis mellifera)
Source: PLoS One. 2013 Sep 5;8(9):e73429. doi: 10.1371/journal.pone.0073429 (PMC3764161; doi:10.1371/journal.pone.0073429)

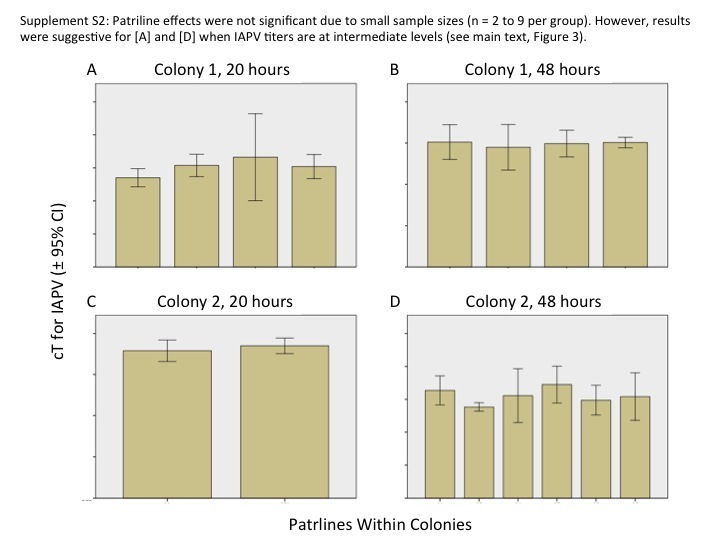

Supplement: Figure S1 — Patriline differences of IAPV titers within colonies suggest a genetic basis for virus resistance in honey bees. (JPG) [file pone.0073429.s001.jpg]
