# Supplementary material for: In Vitro Infection of Pupae with Israeli Acute Paralysis Virus Suggests Disturbance of Transcriptional Homeostasis in Honey Bees (Apis mellifera)
Source: PLoS One. 2013 Sep 5;8(9):e73429. doi: 10.1371/journal.pone.0073429 (PMC3764161; doi:10.1371/journal.pone.0073429)
Supplement: Preliminary Experiments S1 — Two experimental inoculations of a limited number of bees with IAPV suggested that viral infection causes broad-scale alterations of transcript patterns, including immune and reference genes. (PDF) [file pone.0073429.s002.pdf]

S1- Preliminary experiments.

Experiment 01.

A frame of capped brood (worker pupae) was removed from two strong hives at the Bee Research Lab in Beltsville, MD. Early stage pupae with white eyes and a fully formed head were gently removed using fine-tipped forceps and placed onto a hand-molded filter paper rack inside plastic petri dishes (Figure 1) in an incubator ($34^0$C with 80-95% humidity) until inoculation.

One microliter of the inoculant (PBS as control or virus solution containing $10^4$ genome equivalents of IAPV) was manually injected using 10 μL syringe, Model 701 (Hamilton, Nevada). The needle was inserted into the lateral abdomen between the fourth and fifth tergite (Figure 2A).

The first treatment and control groups of bees (n = 25 for each group) were monitored for four days in order to record the time line of visual symptoms and the appropriateness of controls. A subset of control and IAPV inoculated bees were collected during the experimental period and analyzed for gene expression patterns. In this study, an array of immune, developmental, and detoxification genes, viral transcripts, and markers for other honey bee pathogens (Supplement S1) was screened. Pooled RNA from different time points (0, 3, 12, and 36 hours) of the control and the virus-inoculated group were assayed by RT-qPCR, using published methods [39,40].

Experiment 02.

Brood from an additional colony in the same apiary were inoculated and individually analyzed for patterns of expression of a smaller subset of genes. In addition to three samples taken just before the experiment, three control and five treatment bees were sampled at 3 hours, 12 hours, and 36 hours after inoculation. In this experiment, all samples were analyzed as individuals (biological replicates), following the methods described below.

*Experiment 01 and 02 results.*

The screening of transcripts in the pooled samples of the experiment 01 showed an increase of many transcripts in the IAPV infected samples compared to the controls S1-A (Below). The elevated presence of most transcripts in the sample after 12 hours of IAPV infection ($6^{th}$ column)

was particularly pronounced. Of the 40 investigated transcripts, 28 were more abundant in this than in the other six samples. Correspondingly, low Ct values were observed in all transcript classes, including pathogens, immune genes, detoxification, and reference genes.

The screening of six of the 40 transcripts in individual samples from a second colony of the same bee yard also showed higher transcript levels in virus infected pupae S1-B (below). This included the reference gene *Actin*, which led to the evaluation of all data based on Ct-values. Two-factorial ANOVAs revealed significant differences for all genes analyzed except *Eater* (Table 2). Independent of time and treatment effects, *Actin*, *Hymenoptaecin*, and *Eater* are significantly associated with IAPV titers, as measured by ANCOVA (Table S1).

With regards to comparing the multiple time points, Bonferroni post-hoc tests revealed significant differences between the 36 hour samples and all other samples for *Actin*, while *Hymenoptaecin* and *Abaecin* showed a difference in expression between 36 hours and 0 and 3 hours, and *PRPS2* differed between 20 and 36 hours.

These results in two independent experiments indicates a strong disturbance on the transcriptional level that worth to be investigated deeply.

S1: Gene expression patterns in the experiment 01.
[A] Abundance of the initialy screened transcripts in control group(4 leftmost columns), and IAPV inoculated bee pupae. In this experiment a initial screening of genes were conducted using pulled samples (n=6), see details on Material and methods. These transcrips are contained in the Beepath array. See Evans (2006) for discussion of these transcripts.

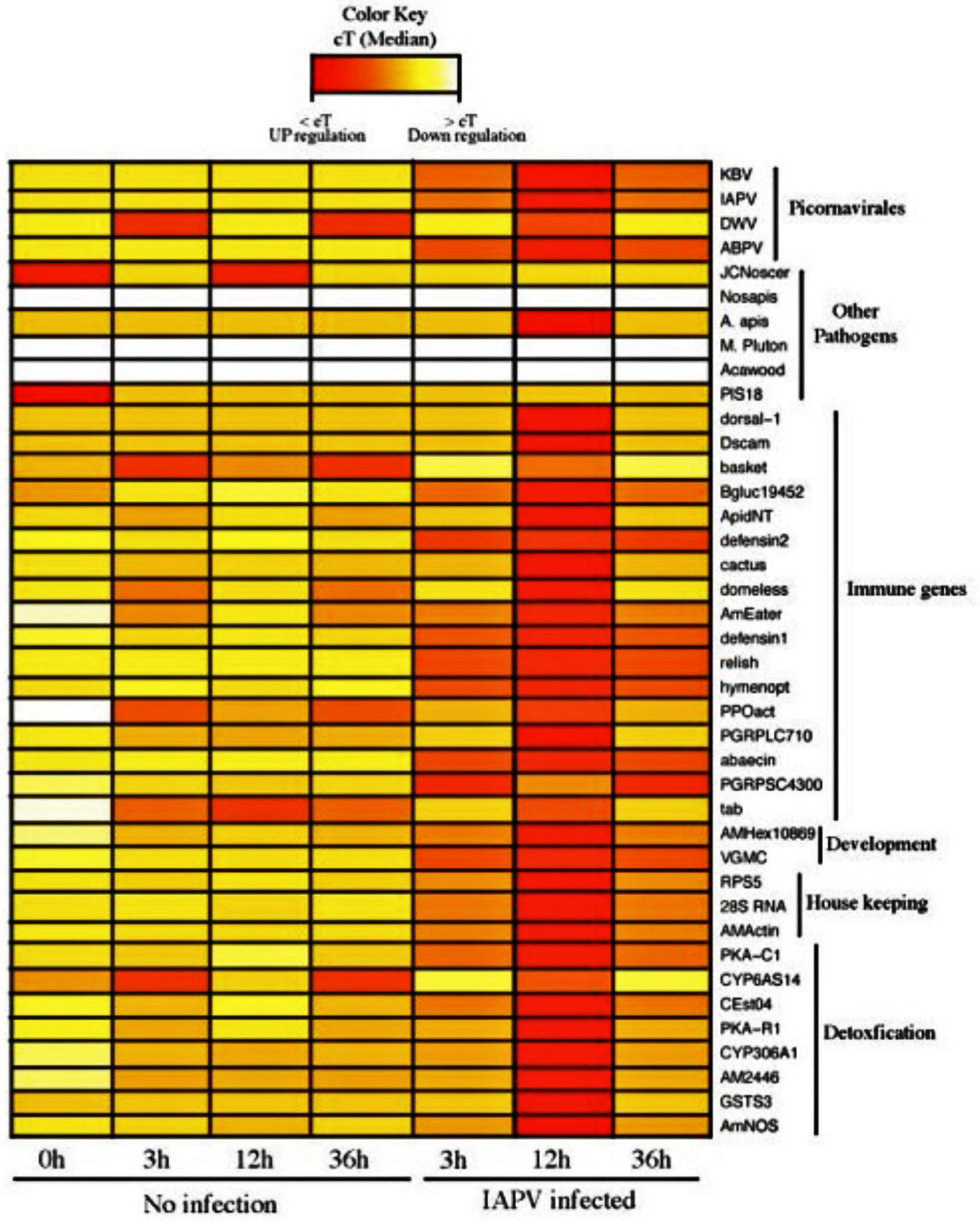

S2: Gene expression patterns in the experiment 02.

A biological replicate of experiment 01 (another hive analyzed) was conducted using some of gene targets from experiment 01. qPCR were conducted using individual bees instead of pulled samples from experiment 01. Control (4 leftmost columns) and IAPV inoculated (3 rightmost columns). Details on Material and Methods.

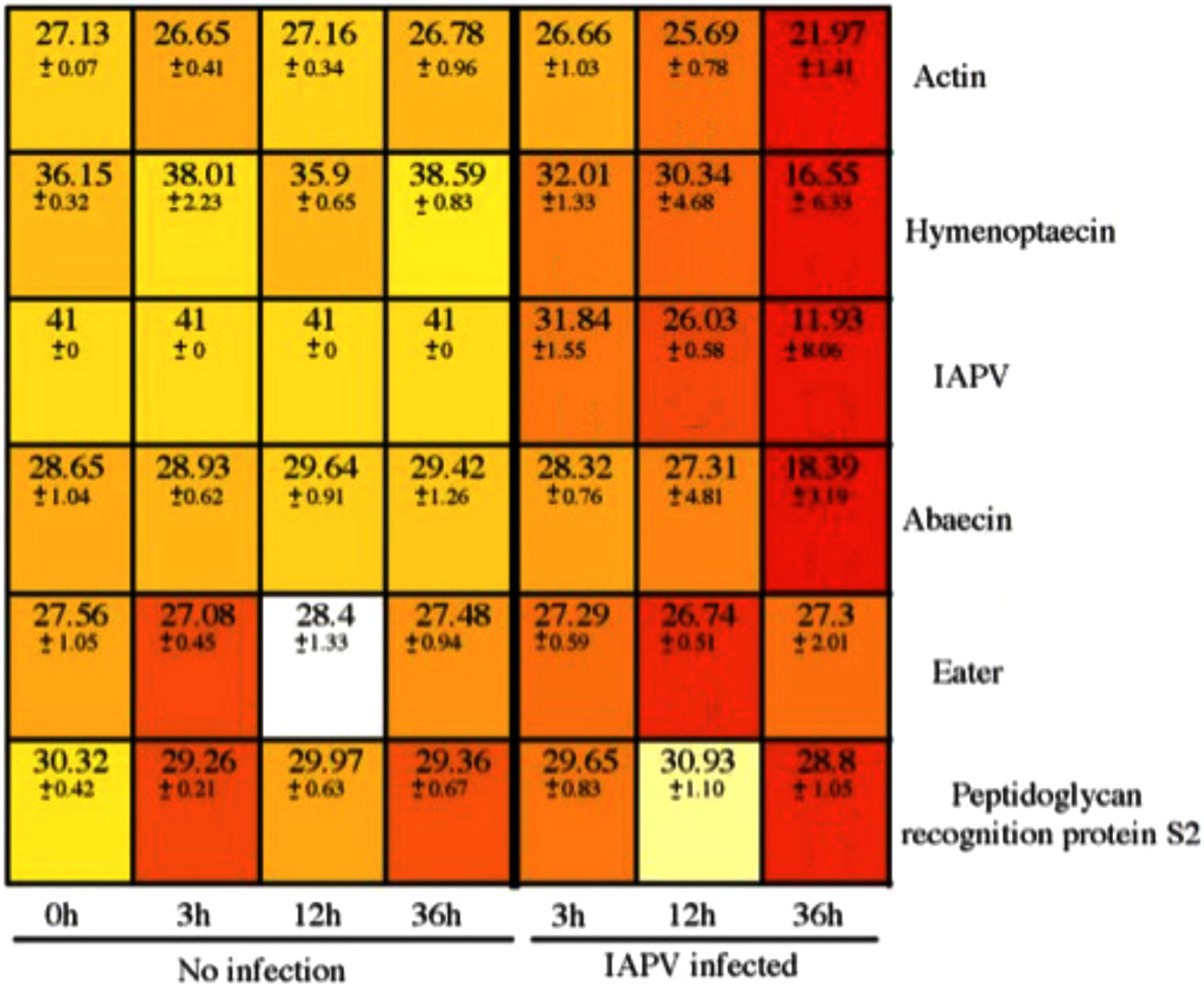

Table S1: IAPV-inoculation (treatment) and timing affected* gene expression in the preliminary experiment 02.

* Significant effects are underlined.

| Gene | Time Effect | Treatment Effect | Interaction | Association with IAPV |
|---|---|---|---|---|
| ***Actin*** | **$F_{(3,20)} = 6.4$**<br>**$P = 0.003$** | **$F_{(1,20)} = 22.7$**<br>**$P < 0.001$** | **$F_{(2,20)} = 7.4$**<br>**$P = 0.004$** | **$\eta_p^2 = 0.23$,**<br>**$p = 0.029$** |
| ***Hymenoptaecin*** | **$F_{(3,20)} = 4.2$**<br>**$P = 0.019$** | **$F_{(1,20)} = 54.6$**<br>**$P < 0.001$** | **$F_{(2,20)} = 7.1$**<br>**$P = 0.005$** | **$\eta_p^2 = 0.40$,**<br>**$p = 0.002$** |
| ***Abaecin*** | **$F_{(3,20)} = 4.1$**<br>**$P = 0.021$** | **$F_{(1,20)} = 17.6$**<br>**$P < 0.001$** | **$F_{(2,20)} = 5.8$**<br>**$P = 0.010$** | $\eta_p^2 = 0.10$,<br>$p = 0.155$ |
| ***Eater*** | $F_{(3,20)} = 0.13$<br>$P = 0.942$ | $F_{(1,20)} = 0.31$<br>$P = 0.582$ | $F_{(2,20)} = 0.94$<br>$P = 0.407$ | **$\eta_p^2 = 0.41$,**<br>**$p = 0.002$** |
| ***PRPS2*** | **$F_{(3,20)} = 3.5$**<br>**$P = 0.036$** | $F_{(1,20)} = 1.0$<br>$P = 0.328$ | $F_{(2,20)} = 1.9$<br>$P = 0.173$ | $\eta_p^2 = 0.02$,<br>$p = 0.559$ |
| **IAPV** | **$F_{(3,20)} = 5.3$**<br>**$P = 0.007$** | **$F_{(1,20)} = 90.2$**<br>**$P < 0.001$** | **$F_{(2,20)} = 7.3$**<br>**$P = 0.004$** | N/A |
