# Supplementary material for: In Vitro Infection of Pupae with Israeli Acute Paralysis Virus Suggests Disturbance of Transcriptional Homeostasis in Honey Bees (Apis mellifera)
Source: PLoS One. 2013 Sep 5;8(9):e73429. doi: 10.1371/journal.pone.0073429 (PMC3764161; doi:10.1371/journal.pone.0073429)
Supplement: Raw Data S1 — Raw Ct values for all qPCRs run in the main experiment. (PDF) [file pone.0073429.s003.pdf]

Table A. Experiment 03-Full qPCR data set (Raw Ct data).

Colony	*Treatment*	*Time-point*	*(Patriline)*	*IAPV (Ct)*	*Actin (Ct)*	*R28S (Ct)*	*R18S (Ct)*	*RPS5 (Ct)*	*mGST1 (Ct)*	*DWV (Ct)*	*Histone (Ct)*
1	IAPV	0h	A	45	26.59651566	17.74129486	21.63273621	30.02298164	25.76039124	45	28.92965126
1	IAPV	0h	A	45	27.13964844	19.53735542	24.59539795	32.29816055	29.41586494	29.92755508	26.52066803
1	IAPV	0h	A16	45	31.37457657	21.38537025	28.75237274	33.97804642	26.55736732	45	26.00699043
1	IAPV	0h	A8	45	25.89570618	19.63869858	22.10761261	29.95853043	28.14509201	45	27.48459053
1	IAPV	0h	A1	45	25.78241158	19.98479271	21.8247261	29.64994431	29.00102234	45	27.17256927
1	IAPV	5h	A10	45	30.16277695	21.28669548	30.18173027	33.12330246	26.25341606	45	30.58750534
1	IAPV	5h	A48	45	31.283741	20.91906357	29.5752697	27.83522987	25.03351974	45	30.88888741
1	IAPV	5h	A	45	31.76287079	21.27567863	26.68290138	34.67322159	31.10067558	45	30.62383461
1	IAPV	5h	A	45	29.47990036	20.49040604	27.31605148	37.9886322	26.81801987	37.1590004	29.27351189
1	IAPV	5h	A44	45	30.27072525	21.72389984	31.10178566	31.35785866	25.95772552	30.40103722	31.45329285
1	IAPV	5h	A44	45	29.40038681	20.59046936	27.4844799	34.278862	29.36560631	45	27.25916862
1	IAPV	5h	A10	45	29.61483192	20.73877335	26.61195755	33.50440216	25.39300156	45	28.54982567
1	IAPV	5h	A10	45	29.61947823	20.33362961	26.69243622	34.04969025	25.98556709	45	27.97181702
1	IAPV	5h	A8	45	30.55885315	22.45968056	30.78660011	35.52729416	26.3739872	45	31.30252457
1	IAPV	20h	A12	24.96791267	24.39276886	18.16262627	23.7035923	21.16989326	21.70749092	45	23.60948563
1	IAPV	20h	A6	32.18207932	24.68832016	21.32090378	28.54394341	25.08119392	25.26978111	45	27.63312912
1	IAPV	20h	A4	26.90733719	24.4712677	18.33004761	25.66243553	23.42463875	23.89171982	45	25.93608475
1	IAPV	20h	A5	33.408741	25.46068001	19.09882355	32.82258224	24.61623383	22.15780449	38.25573349	26.43421173
1	IAPV	20h	A21	29.86983871	25.51852036	18.34225845	27.33825493	24.86735916	24.15517044	37.27670288	26.86917877
1	IAPV	20h	A35	30.71754837	24.88805962	16.2258873	19.5415802	25.28294182	24.92617035	36.82174683	27.41194916
1	IAPV	20h	A2	31.00568771	26.28848457	20.49542809	27.5388031	25.81524658	24.87802124	45	27.34676552
1	IAPV	20h	A8	29.43145752	25.51431847	20.31785011	27.53884125	24.46337509	24.25037384	45	27.21218109
1	IAPV	20h	A8	27.73806953	25.33732796	19.79667854	27.44761848	22.39690971	21.69755745	38.08959961	24.53247452
1	IAPV	20h	A12	31.94789124	26.27253723	20.21626854	28.19609261	26.02178955	24.91955757	45	28.46097565
1	IAPV	20h	A8	32.95851517	27.06611252	19.7997818	29.59944725	26.90154266	24.53845978	45	30.35840225
1	IAPV	20h	A12	25.24282837	25.67242241	18.50145912	23.19584656	23.3171196	22.56938171	45	25.67084885
1	IAPV	20h	A12	27.68117905	24.12569618	19.34441376	25.57274246	22.03248787	22.13539696	38.24210739	24.49154091
1	IAPV	20h	A2	31.26774788	25.57361794	19.81160164	28.24251556	25.65459061	24.43452454	39.36383438	27.34363365
1	IAPV	20h	A12	25.59926605	23.81503677	18.84762573	23.74634552	22.05360603	21.99630547	32.3869133	24.93622589
1	IAPV	20h	A8	31.45365524	25.66424751	20.22660255	29.00309753	25.66288567	24.27244377	30.8552494	28.52570724
1	IAPV	20h	A2	32.8680954	26.06418419	21.31746292	28.83196259	27.2573185	25.67023659	45	28.23671532
1	IAPV	20h	A2	27.80626106	25.75393295	18.78435326	26.38419533	22.15158653	22.08806229	38.74574661	24.76153183
1	IAPV	20h	A22	26.92821693	25.49269104	19.48087502	25.78831482	22.69810867	22.98109818	45	25.28488541
1	IAPV	20h	A12	26.69201469	23.96993637	18.80877495	24.51828957	22.7608242	22.55991936	45	24.97377968
1	IAPV	20h	A6	34.25333023	26.80828094	20.64209366	29.82148933	26.49649429	24.71011925	45	28.43971825
1	IAPV	48h	A14	28.78945732	25.44936943	19.13240242	25.70998192	26.29626846	25.58679581	36.33451462	29.9510231
1	IAPV	48h	A1	29.79759026	27.22170067	20.69077492	27.73846436	27.28600693	25.9091568	45	28.8322258
1	IAPV	48h	A24	31.97141838	26.22608566	19.84484863	29.10721016	27.08732605	26.39024544	39.25255203	29.5283699
1	IAPV	48h	A14	28.24080276	25.58334541	18.95674706	25.71064758	26.08225632	25.87759972	23.35531044	29.47028732
1	IAPV	48h	A16	28.82387733	25.3948555	19.17998123	29.1243515	26.81931496	24.54695129	45	28.01258659
1	IAPV	48h	A16	25.95560837	24.65517807	18.43475151	27.64063454	25.88802338	24.72005081	45	28.59257507
1	IAPV	48h	A16	32.05822372	25.73745918	19.37097359	29.13945007	25.99730301	25.49061775	45	28.81838799
1	IAPV	48h	A16	32.26318359	25.71261024	19.66135406	30.10786629	26.17975807	24.97955894	33.34578705	30.68997765
1	IAPV	48h	A14	22.12029266	25.13150978	18.16818428	24.74819946	25.49166679	24.89219284	32.76569748	28.33789444
1	IAPV	48h	A16	30.30620956	25.31488609	18.99665451	28.01311684	25.54183769	24.74493027	45	28.31669426
1	IAPV	48h	A22	30.39677811	25.44996262	19.74729729	28.62542152	25.73612976	24.65803528	45	28.51745987
1	IAPV	48h	A1	28.57409096	26.35684967	18.58552551	25.8229332	26.96168327	26.18490982	45	30.03858376
1	IAPV	48h	A22	30.56142807	24.95923424	19.68944359	27.93379021	25.9690609	25.24988174	45	27.7490406
1	IAPV	48h	A1	28.49464607	25.36740303	18.94305801	26.28373528	25.21094513	24.70283127	45	27.99329567
1	IAPV	48h	A14	32.25779724	26.65611267	20.77882957	28.19628716	26.94119453	26.34264755	45	29.61595345
1	IAPV	48h	A14	33.50438309	25.46031761	20.72481728	30.72210312	27.366045	24.36535835	45	28.44104767
1	IAPV	48h	A22	28.95034218	26.09595108	19.19752312	26.56457901	25.86887932	25.0651207	45	29.92144585
1	IAPV	48h	A1	34.10787201	27.64983368	19.28584671	27.20988464	29.21523285	27.32551765	45	30.22303963
1	IAPV	48h	A22	30.63644791	25.78874969	19.80685997	27.87696648	25.43936157	25.11822319	45	29.19617844
1	PBS	0h	A	45	28.02779007	21.21423531	25.11334038	31.80042648	24.70139694	37.72607422	27.10432053
1	PBS	0h	A22	45	27.21440125	20.30673981	22.27492523	29.64528656	31.21571922	45	25.97267723
1	PBS	0h	A12	45	27.40592194	19.80152512	21.86914825	30.00285721	30.77766037	45	25.58716774
1	PBS	0h	A	45	26.24214363	20.68218613	22.5585804	29.8098011	29.58679581	45	27.7855072
1	PBS	0h	A14	45	24.84436226	18.9182148	21.17214584	28.72660446	29.35190392	45	26.74208641
1	PBS	5h	A	45	30.33633995	22.94524765	26.00469398	32.4796257	25.04172134	28.69168663	30.02124977
1	PBS	5h	A	45	28.67584229	22.23257828	24.3851223	31.37657547	25.72645187	45	28.42461777
1	PBS	5h	A	45	29.13241768	21.00162506	24.29091263	31.11575127	24.48933983	45	29.18480682
1	PBS	5h	A	45	29.38555908	22.58969688	24.37712669	31.22954178	26.2868557	45	28.07213211
1	PBS	5h	A	45	27.61459923	20.5433712	22.22904778	31.062603	25.35367966	45	28.17544556
1	PBS	20h	A21	45	28.11748695	20.96188736	26.59068298	27.99094963	25.91049767	32.75988007	28.34764481
1	PBS	20h	A12	45	32.79116821	20.52939987	23.98424721	23.98010063	23.02620888	45	25.67113686
1	PBS	20h	A1	45	28.52720261	20.73923111	25.16427803	30.57357597	23.97019958	34.81875992	27.69230843
1	PBS	20h	A	45	27.65568542	20.10699844	24.26889038	31.51585579	25.63031197	35.32347107	26.27880096
1	PBS	20h	A5	45	26.77435112	18.41233826	20.33146477	29.27518272	25.24497223	30.76049995	25.53681374
1	PBS	20h	A14	45	29.90848541	20.5766468	25.40504837	31.13480759	23.73629379	45	29.65769196
1	PBS	48h	A	45	29.56162262	23.1196537	28.23685646	33.94851685	29.83434486	32.75988007	31.93861771
1	PBS	48h	A	45	28.72618103	20.41880989	27.38043594	29.80668449	27.28353882	45	27.59048653
1	PBS	48h	A	45	30.83903503	23.15721893	29.23399162	34.90407181	28.26594925	34.81875992	29.25412941
1	PBS	48h	A	45	28.60655785	21.05953217	30.76358414	29.29448128	27.12375641	35.32347107	29.8705864
1	PBS	48h	A	45	27.37637901	18.87007141	21.42494583	30.4570694	27.68270683	30.76049995	26.41038132
1	PBS	48h	A3	45	26.82799149	18.98023415	22.41392136	30.67421341	26.52070427	45	25.16576576
1	W0	0h	A	45	30.52975464	20.23757172	24.35189438	31.69368553	26.02574348	45	29.7389164
1	W0	0h	A	45	29.15894318	20.96964073	25.51231956	28.62093544	25.48889351	45	29.21031761
1	W0	0h	A	45	28.8986187	19.20507431	24.97111893	32.76019287	26.00584221	27.25421143	29.08285522
1	W0	0h	A	45	26.51194191	20.48766899	22.30076981	29.77986336	28.18572617	45	28.34083176
1	W0	0h	A4	45	27.07718086	21.46161461	23.44748688	31.16217804	25.19530487	45	28.63464737
1	W0	5h	A48	45	29.60900688	21.65260315	32.45973587	30.37434578	27.35723114	30.44621849	34.14476013
1	W0	5h	A5	45	26.97587395	17.71082306	26.58615685	25.39469528	26.19352913	45	28.89962959
1	W0	5h	A44	45	28.74389648	19.87309837	25.20380402	32.75033569	26.33598518	31.97112274	27.85525131
1	W0	5h	A4	45	25.34540367	19.51522255	21.73686981	29.27418327	26.93639755	45	26.74129486
1	W0	5h	A5	45	25.98820686	19.86831856	21.6837101	29.39710045	29.62609863	45	28.15633202
1	W0	20h	A18	45	32.71434784	21.6918354	29.42516899	35.24028015	26.27397156	45	31.8314991
1	W0	20h	A14	45	32.37949753	22.6340332	33.47364426	34.421772	26.73730469	45	32.78145981
1	W0	20h	A22	45	32.37705994	21.91	32.95721817	32.53335953	27.82349586	30.84788704	34.97551727
1	W0	20h	A	45	32.46720505	21.91	29.97118568	36.92222595	28.08038139	45	33.11588669
1	W0	20h	A	45	32.60459137	21.91	32.24419785	36.08023071	28.31804085	45	34.08880997
1	W0	20h	A	45	32.03098297	21.38484001	27.74443054	35.52666092	26.92219734	45	29.53760338
1	W0	20h	A	45	32.29292679	21.94197845	30.0874958	34.36837769	26.44176674	36.40375519	34.31414032
1	W0	20h	A	45	32.46580887	21.91	29.97914886	36.36956024	26.65849304	45	34.93217468
1	W0	48h	A47	45	29.88254166	21.06987572	27.9197464	27.54570961	26.14486504	45	29.89002419
1	W0	48h	A16	45	28.91262054	21.29145622	29.50152588	27.21520996	25.11152458	45	30.21789169
1	W0	48h	A4	45	29.76469612	21.82671928	29.04380798	28.26239586	25.39554977	45	30.44716644
1	W0	48h	A19	45	30.37425041	20.82560349	25.53015137	35.94176102	26.72872734	45	30.58832741
1	W0	48h	A5	45	28.2421875	19.8273983	21.62683105	30.77724266	28.16323662	45	28.10962296
2	IAPV	0h	A3	45	27.28992462	21.17812157	23.59119797	29.76473618	25.67866325	45	26.2976284
2	IAPV	0h	A28	45	28.27864838	20.02962875	27.11003876	29.96159744	26.53633308	45	27.313694
2	IAPV	0h	A2	45	28.72435188	20.75210571	25.03322411	32.6952858	24.68649673	45	27.0988102
2	IAPV	0h	A24	45	25.90169525	20.28705406	22.39563751	29.90403366	30.0980835	45	27.64941978
2	IAPV	0h	A11	45	29.76956367	18.77061081	20.34890175	29.00238228	29.30986595	45	26.90237808
2	IAPV	5h	A4	45	31.14331245	21.25048447	30.15968895	35.33185577	26.92002869	45	34.5616188
2	IAPV	5h	A8	38.75836563	27.6737709	20.91810799	25.33917999	27.84200287	22.75868034	38.60665131	26.62392235
2	IAPV	5h	A10	45	28.52352715	20.25513268	25.87747765	31.74294281	25.86233902	28.87318802	27.50723839
2	IAPV	5h	A4	45	31.72908974	21.47603035	28.52936363	34.57462311	24.82016563	45	29.90494728
2	IAPV	5h	A11	39.07796097	27.79738617	21.86368561	24.97456741	28.3627739	24.31364059	37.86790848	26.33769035
2	IAPV	5h	A	45	27.41127968	19.60339165	24.37579727	31.16674805	25.75426674	45	26.7712574
2	IAPV	20h	A18	28.59368134	24.88949966	18.77304268	24.92965317	25.06338882	24.51736832	38.42314529	29.72563171
2	IAPV	20h	A9	30.14294243	25.83745193	18.92807007	26.75944138	25.58395386	25.03590775	45	27.00163651
2	IAPV	20h	A37	37.61975861	25.82002449	19.60587463	27.75257874	25.88843346	24.34768867	45	27.80627251
2	IAPV	20h	A	31.57342339	26.39573097	19.039608	28.19422722	25.58341026	25.08426857	45	29.18491745
2	IAPV	20h	A	38.63586807	26.17854881	16.47409058	21.00334358	27.09218788	25.028368	45	29.65770912
2	IAPV	20h	A21	38.83010864	26.60935974	21.69389725	30.82739067	27.95895191	25.32771111	45	30.17360115
2	IAPV	20h	A	38.83010864	29.32020187	22.27572441	32.25288773	26.68270874	23.95244217	45	29.3558197
2	IAPV	20h	A1	33.88871384	25.24965858	20.33517265	28.70541954	26.80421066	24.5871582	45	28.03005409
2	IAPV	20h	A1	38.00684738	27.47684097	21.22460938	29.24366379	27.20778275	25.05468369	45	29.73099709
2	IAPV	20h	A1	33.68357849	25.84479904	19.242939	26.88510704	26.42162895	24.43775558	45	27.77210236
2	IAPV	20h	A1	38.92809949	28.54199028	19.87637138	30.57339859	28.68340683	25.82117271	45	29.39431953
2	IAPV	20h	A1	38.7298769	28.68892288	20.37159729	29.88269043	29.07884789	25.84190178	45	29.70499992
2	IAPV	20h	A1	31.57342339	26.07491684	20.72212791	33.33343174	31.12971878	24.59385109	45	30.39792061
2	IAPV	20h	A21	35.46301723	28.47579384	20.80975914	32.56902313	32.11119843	25.42036057	45	32.95755768
2	IAPV	20h	A1	39.22463226	28.23076057	19.22953415	28.76862717	31.18702316	25.42732239	45	30.6378479
2	IAPV	20h	A21	35.46301723	28.55725479	21.35611153	33.34307861	34.93157959	25.84423637	45	33.72541428
2	IAPV	20h	A21	38.0292511	27.35211372	23.59540558	30.5524807	27.17694547	27.23350143	45	30.48626518
2	IAPV	20h	A51	35.10198975	27.61785889	21.45199394	32.29853821	27.91359138	24.2114048	45	28.85785675
2	IAPV	20h	A1	37.55179977	27.32303238	19.97234917	31.53600693	29.47927666	25.81834221	45	31.86992264
2	IAPV	20h	A21	37.13684082	26.96992874	22.68699074	32.25242615	29.91158867	26.97751617	26.79151344	33.15707397
2	IAPV	20h	A	33.19203567	26.88424301	20.41428772	30.99032974	26.7448864	24.3637352	45	28.90121651
2	IAPV	20h	A1	30.59558105	28.13938904	22.38731571	32.16333389	29.84820938	25.80405998	45	30.91521072
2	IAPV	20h	A11	34.33229828	26.63992031	20.43973732	29.58203316	27.25756645	25.05240631	33.2927475	30.59500885
2	IAPV	48h	A1	35.50136566	27.6055088	23.47616959	34.2466011	27.53121758	24.65140533	45	29.35431862
2	IAPV	48h	A2	27.64784622	27.44649124	17.06181145	25.18787766	24.65714836	24.41002464	45	25.42156601
2	IAPV	48h	A4	34.22333908	26.68011665	20.65477943	30.54034042	28.570158	25.97505569	35.13706207	30.27414322
2	IAPV	48h	A5	35.45132446	27.91725922	23.06275749	32.32239532	27.91964149	24.57351494	45	29.39671326
2	IAPV	48h	A1	33.25637817	25.88813019	21.32721519	31.41445541	27.47567368	25.24139786	45	29.78920555
2	IAPV	48h	A6	24.95273018	25.95221138	16.33724213	23.71800804	24.36170387	23.91131592	45	25.1382885
2	IAPV	48h	A6	34.45065689	27.81661606	21.36802292	30.43552971	27.26685715	24.71763611	45	29.36093903
2	IAPV	48h	A6	29.99205589	28.24917984	17.40148735	26.41011047	25.93785095	26.88577652	45	27.22488785
2	IAPV	48h	A8	31.36209869	26.40092468	19.76861572	27.64454651	25.91171455	24.34650421	45	31.62717438
2	IAPV	48h	A5	39.02220917	28.99126434	21.19329262	29.76803207	29.53314781	25.5450325	45	30.90889931
2	IAPV	48h	A2	27.15677071	27.12752724	16.40039063	23.69550323	24.26776314	24.92378998	45	25.13785553
2	IAPV	48h	A2	27.41435814	26.80802155	16.26488113	23.63394165	24.40624428	24.9325943	45	25.94699097
2	IAPV	48h	A5	31.37314034	26.76209831	21.12623978	29.7227993	26.06127167	23.65803337	45	29.45194054
2	IAPV	48h	A6	31.59365654	27.38658714	20.37339211	30.2646122	26.2207489	23.95186424	45	28.59966278
2	IAPV	48h	A4	36.18881607	29.36986732	16.20320129	18.76062393	32.96772003	31.53878212	45	27.70708084
2	IAPV	48h	A8	30.22664642	26.37873077	19.48325348	27.66617966	25.87179947	24.3967762	45	28.17721748
2	IAPV	48h	A6	27.57705688	27.36164856	16.86766624	24.78939247	24.98842239	24.83398056	45	25.38884926
2	IAPV	48h	A4	29.29595757	27.31689262	18.94795609	27.6215744	24.41277313	25.1498642	45	25.96108437
2	IAPV	48h	A1	28.88447189	26.09274673	20.95438194	29.78646088	25.69316483	25.19164658	45	28.5237484
2	IAPV	48h	A5	32.04553986	26.95773506	22.25563812	29.49111557	25.88865089	24.32752609	35.50148773	29.61608124
2	IAPV	48h	A2	29.41596794	28.22192001	16.89108658	25.68241882	24.52149391	26.01830864	45	26.94630814
2	IAPV	48h	A1	33.09880447	26.15282249	20.62906647	30.8101387	27.81924438	26.375494	45	31.3406868
2	IAPV	48h	A2	26.63442421	26.73145676	16.24475479	23.55760765	24.95862579	24.35448265	45	25.24628258
2	IAPV	48h	A4	24.69023514	26.47089386	18.16266251	25.40199089	24.46741867	24.29714775	45	25.34227753
2	PBS	0h	A	45	26.17917824	20.1569767	22.36314583	30.08142853	28.47562408	45	27.35040855
2	PBS	0h	A13	45	26.91013718	20.63319778	22.98787117	31.29089165	24.94595146	45	27.7676239
2	PBS	0h	A38	45	29.55206299	21.83406448	26.30218887	28.91723824	25.34937859	45	30.17640114
2	PBS	0h	A	45	28.40651321	24.51524734	24.83234215	30.78969765	27.53338432	45	27.21395874
2	PBS	0h	A39	45	28.26093864	20.53314018	26.54137802	31.92059898	27.29224968	45	27.53416252
2	PBS	5h	A7	45	26.02630234	19.70051575	22.01345825	30.45311546	28.68039894	45	27.51770401
2	PBS	5h	A12	45	26.59988976	20.6419735	23.2140522	30.48505211	30.67389679	45	27.95218658
2	PBS	5h	A2	45	28.48258972	22.34509468	37.14637756	31.12152672	24.99419212	45	27.35988235
2	PBS	5h	A22	45	28.25401688	20.66889191	24.70906448	31.6289444	25.32865715	45	26.46139145
2	PBS	5h	A6	45	27.78141785	21.90336609	24.93609619	31.46282959	27.19542313	45	28.96212387
2	PBS	20h	A19	45	26.24106598	20.72164154	23.88546562	26.50592804	24.64399719	45	28.35520554
2	PBS	20h	A21	45	28.74697685	21.30938148	27.16926003	33.86141586	27.55992126	29.27152061	27.2311306
2	PBS	20h	A8	45	28.68318558	22.5582428	31.01281548	26.68611145	23.85966682	26.57775307	28.92381096
2	PBS	20h	A21	45	28.21011734	20.95669746	26.22075462	31.99298477	26.07149315	33.35804749	26.54658699
2	PBS	20h	A21	45	26.6861248	19.4224205	21.43088913	30.27623749	27.04129028	33.23378372	25.24928284
2	PBS	20h	A10	45	32.46326065	24.958004	25.82596016	32.89986801	29.11847305	45	26.45023727
2	PBS	48h	A4	45	32.18090057	21.57696915	27.97528267	29.08453369	31.39577675	26.70595554	31.61096001
2	PBS	48h	A4	45	29.69252586	20.00762177	24.72283173	26.73221016	26.33431435	45	26.74779701
2	PBS	48h	A2	45	26.81604576	18.00009727	20.6525134	25.88134193	24.45113373	38.79183578	26.74779701
2	PBS	48h	A	45	30.33003998	20.44475989	27.60294151	27.83155981	27.85349846	24.80727386	29.64653587
2	PBS	48h	A4	45	30.09627342	21.0756073	29.13181877	27.91695976	28.82002068	24.68944931	29.77659607
2	PBS	48h	A4	45	30.78600883	19.9907341	25.70861816	26.87057114	27.51813698	25.5415535	31.37146759
2	PBS	48h	A2	45	29.56220055	20.40607643	28.34787996	25.84341049	29.68645668	24.83409691	28.16621208
2	PBS	48h	A	45	28.76045227	18.72967148	23.37256432	25.62865257	24.2471714	45	26.83743668
2	PBS	48h	A6	45	27.5771389	18.18853569	21.19928169	24.96593094	24.22845459	20.97628403	27.25141907
2	PBS	48h	A6	45	30.75703621	20.27140999	26.93176079	26.18537521	26.81282806	24.63622856	28.33342361
2	PBS	48h	A2	45	29.26016617	20.71727943	26.65307426	25.93138403	25.44020081	45	27.43344688
2	PBS	48h	A2	45	28.81259155	19.7362442	25.13791656	25.60902977	24.76107597	45	26.90233421
2	W0	0h	A31	45	27.90324593	19.48304367	20.60112953	29.93394661	27.60212898	30.38757133	28.05654526
2	W0	0h	A21	45	28.76358223	21.46168137	23.80206871	31.60607529	24.93615913	45	28.25424004
2	W0	0h	A35	45	29.24655914	21.60550499	24.23439026	31.91048622	26.06441498	45	29.43838692
2	W0	0h	A20	45	25.9327507	19.15307426	20.86666298	29.34082985	30.25191498	45	27.08689499
2	W0	0h	A3	45	26.36481857	19.83036804	22.48468018	30.66139412	26.10238266	45	28.35152245
2	W0	5h	A12	45	25.46622467	19.65162086	21.54938316	29.76786613	27.38050461	45	27.37620735
2	W0	5h	A11	45	27.03616714	19.95779419	22.31196594	30.34107971	25.41092491	45	27.74962425
2	W0	5h	A1	45	26.59744835	21.42126465	24.32073975	30.92282867	23.38167381	45	27.97363853
2	W0	5h	A22	45	27.60948181	21.13238335	24.32409668	31.72481155	25.2286129	45	28.56311989
2	W0	5h	A2	45	27.73942947	20.18808746	23.82228088	31.33649254	26.6646843	28.77775764	28.62325859
2	W0	20h	A12	45	27.16868782	20.1620388	23.34354591	30.88637543	25.76700211	35.98831558	28.34877014
2	W0	20h	A12	45	28.0947094	19.89229202	23.94269371	31.70282745	27.15576744	45	29.80929375
2	W0	20h	A8	45	26.5982399	19.6486454	22.51175117	29.94697571	29.11866379	45	28.56487465
2	W0	20h	A6	45	27.52121353	19.56697083	23.01508141	31.15841484	24.86211967	45	29.22112656
2	W0	20h	A5	45	28.00038528	20.62117195	25.80946159	33.88284683	24.52898407	45	30.76786232
2	W0	48h	A20	45	27.64068794	19.20203781	22.14946747	31.30548096	28.57611275	45	29.49198532
2	W0	48h	A20	45	25.9077301	19.32519531	21.21355629	29.71589279	29.64064407	45	27.39564133
2	W0	48h	A22	45	25.94681549	19.20109558	21.65468025	29.98515701	29.05694008	45	26.86355972
2	W0	48h	A	45	26.75159645	19.57918358	22.27472687	30.30641174	28.66603279	45	30.32661438
2	W0	48h	A16	45	26.56574059	19.6624279	22.36830902	30.96644783	29.42764282	45	28.07704163
