# Supplementary material for: In Vitro Infection of Pupae with Israeli Acute Paralysis Virus Suggests Disturbance of Transcriptional Homeostasis in Honey Bees (Apis mellifera)
Source: PLoS One. 2013 Sep 5;8(9):e73429. doi: 10.1371/journal.pone.0073429 (PMC3764161; doi:10.1371/journal.pone.0073429)
Supplement: Results S1 — The expression of genes evaluated by full, 3-factorial ANOVAs indicated that developmental time is a significant factor, that was as important as treatment but for most genes interacted with treatment. (PDF) [file pone.0073429.s004.pdf]

S3: Post-hoc tests results for time effects in the main experiment. Shown are average cT values (representing an <u>inverse</u> measure of gene expression) at the different time points without accounting for other factors.  (* = $p < 0.05$, ** = $p < 0.01$, *** = $p < 0.001$)

*Actin*:

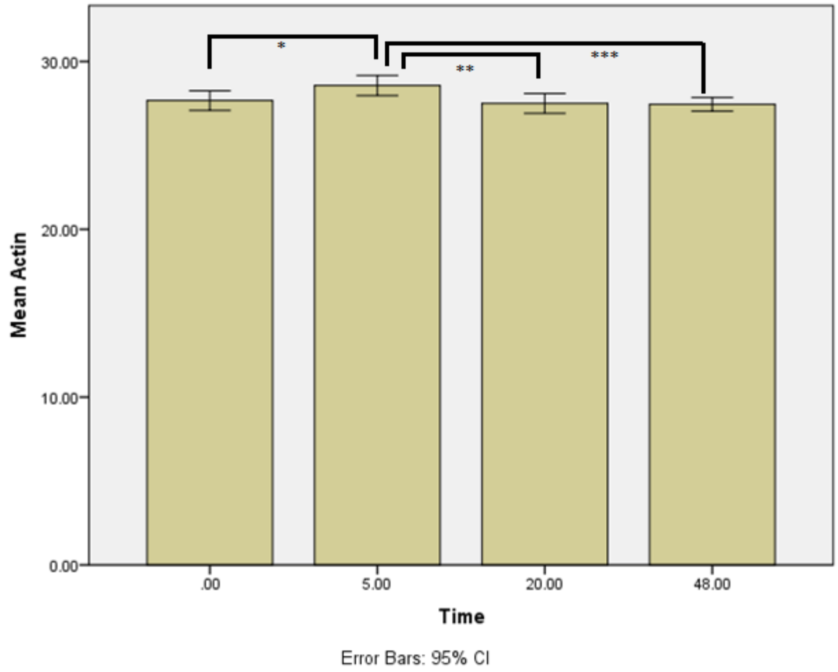


*28S rRNA:*

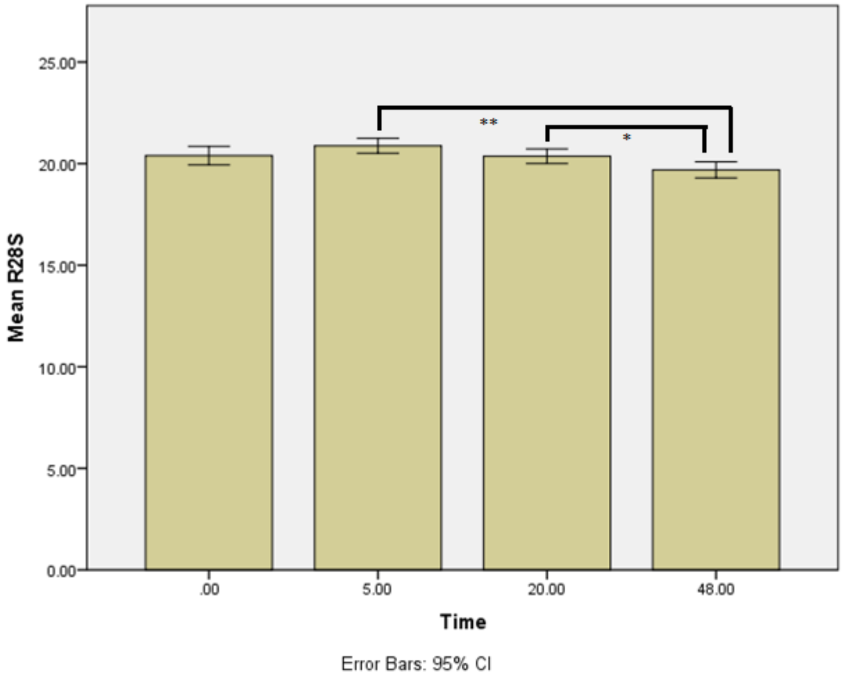

*18S rRNA*:

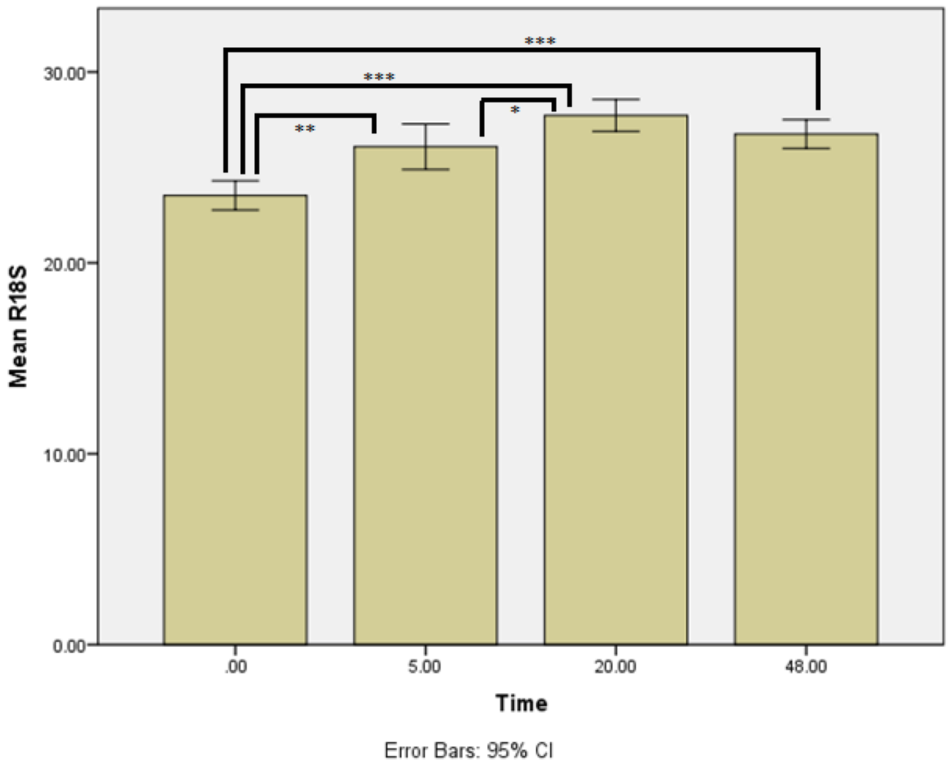


*RPS5*:

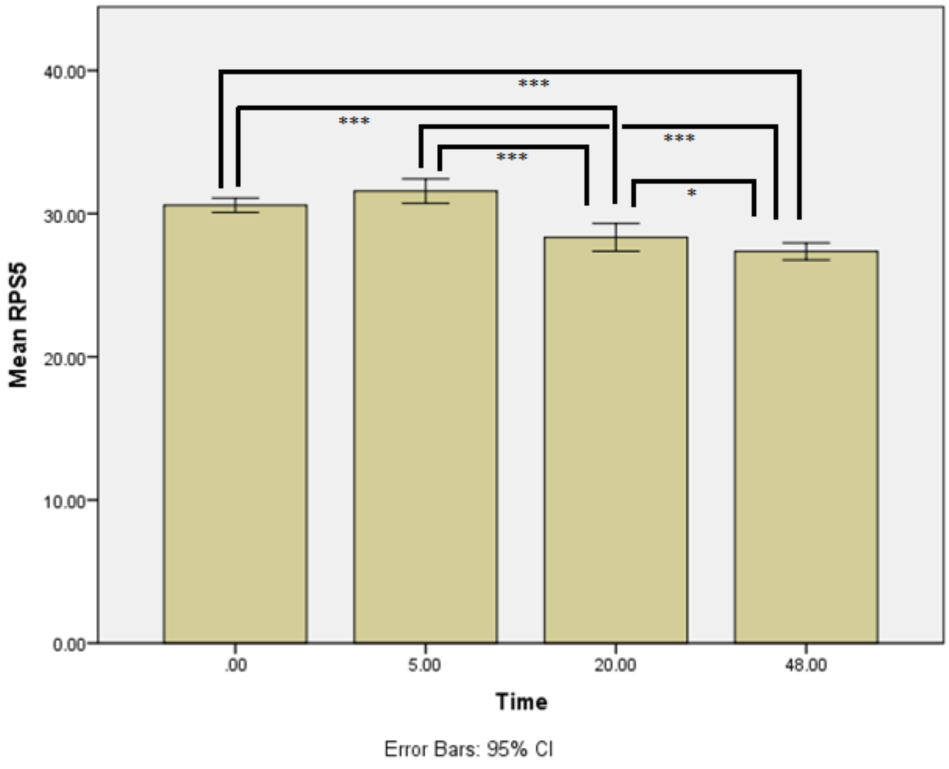

*mGST1:*

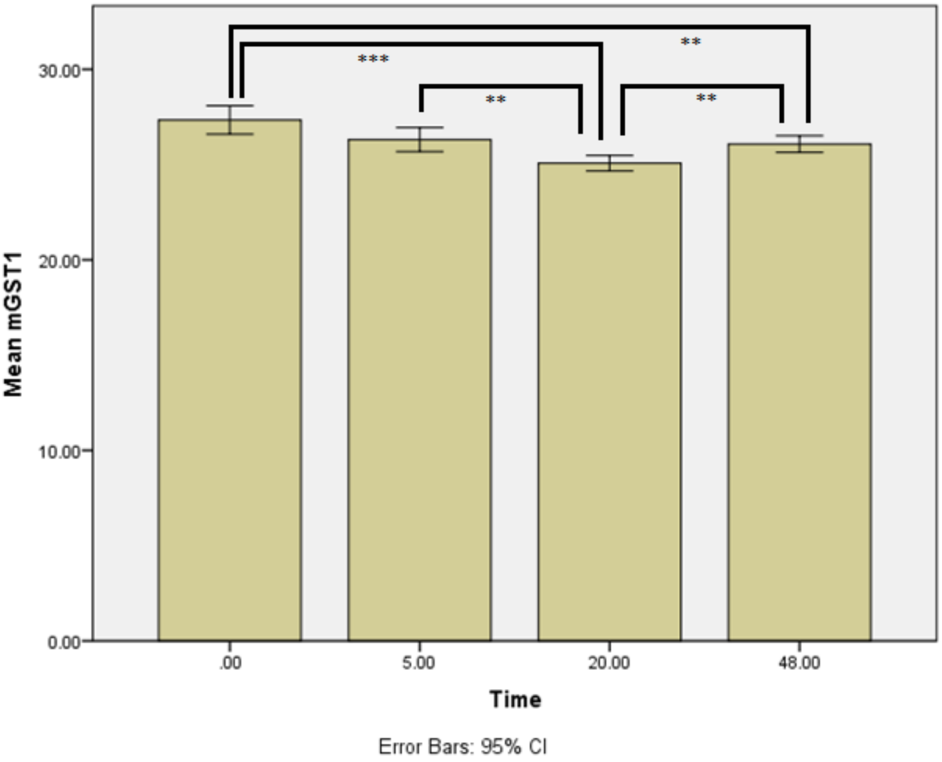


*Histone H2A:*

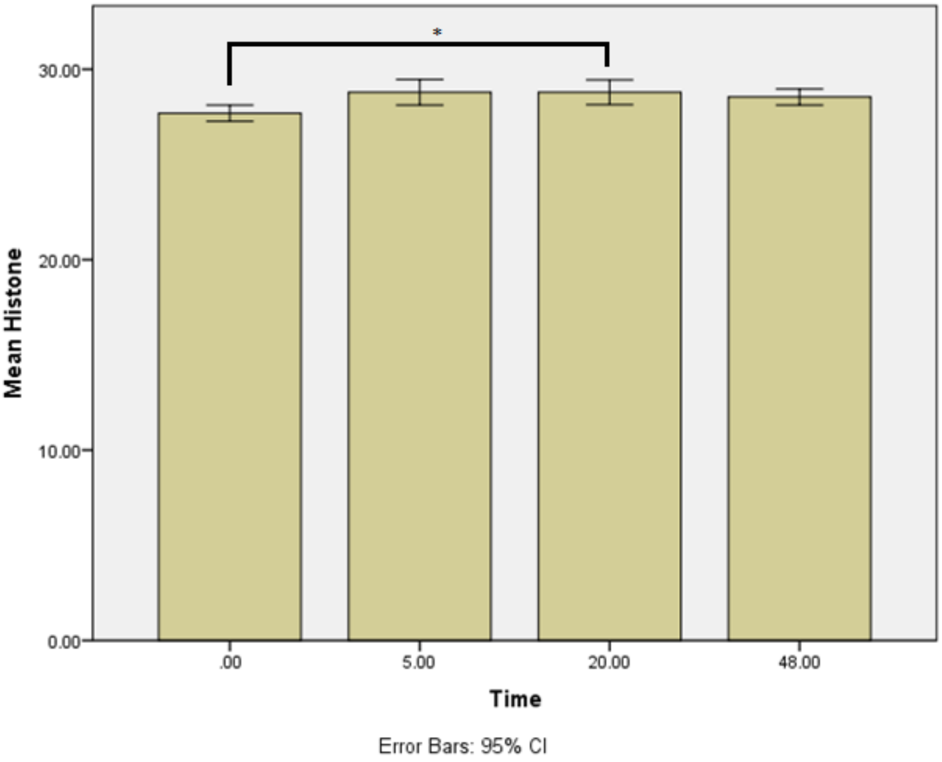

S3: Full results of the 3-factorial ANOVAs of the main experiment

**Tests of Between-Subjects Effects**

Dependent Variable: Actin

| Source | Type III Sum of Squares | df | Mean Square | F | Sig. |
|---|---|---|---|---|---|
| Corrected Model | 547.783[a] | 23 | 23.817 | 15.561 | .000 |
| Intercept | 120094.506 | 1 | 120094.506 | 78463.235 | .000 |
| Treatment | 25.952 | 2 | 12.976 | 8.478 | .000 |
| Time | 11.635 | 3 | 3.878 | 2.534 | .058 |
| Colony | 11.424 | 1 | 11.424 | 7.464 | .007 |
| Treatment * Time | 189.580 | 6 | 31.597 | 20.644 | .000 |
| Treatment * Colony | 57.479 | 2 | 28.740 | 18.777 | .000 |
| Time * Colony | 14.866 | 3 | 4.955 | 3.238 | .023 |
| Treatment * Time * Colony | 61.708 | 6 | 10.285 | 6.719 | .000 |
| Error | 277.036 | 181 | 1.531 | | |
| Total | 158000.152 | 205 | | | |
| Corrected Total | 824.819 | 204 | | | |

a. R Squared = .664 (Adjusted R Squared = .621)

**Tests of Between-Subjects Effects**

Dependent Variable: R28S

| Source | Type III Sum of Squares | df | Mean Square | F | Sig. |
|---|---|---|---|---|---|
| Corrected Model | 132.816[a] | 23 | 5.775 | 3.070 | .000 |
| Intercept | 63932.290 | 1 | 63932.290 | 33992.868 | .000 |
| Treatment | 21.376 | 2 | 10.688 | 5.683 | .004 |
| Time | 16.934 | 3 | 5.645 | 3.001 | .032 |
| Colony | .138 | 1 | .138 | .073 | .787 |
| Treatment * Time | 14.865 | 6 | 2.477 | 1.317 | .252 |
| Treatment * Colony | 8.054 | 2 | 4.027 | 2.141 | .120 |
| Time * Colony | 12.378 | 3 | 4.126 | 2.194 | .090 |
| Treatment * Time * Colony | 22.045 | 6 | 3.674 | 1.954 | .075 |
| Error | 340.417 | 181 | 1.881 | | |
| Total | 84321.653 | 205 | | | |
| Corrected Total | 473.232 | 204 | | | |

a. R Squared = .281 (Adjusted R Squared = .189)

**Tests of Between-Subjects Effects**

Dependent Variable: R18S

| Source | Type III Sum of Squares | df | Mean Square | F | Sig. |
|---|---|---|---|---|---|
| Corrected Model | 1030.639[a] | 23 | 44.810 | 5.773 | .000 |
| Intercept | 99663.715 | 1 | 99663.715 | 12840.455 | .000 |
| Treatment | 142.618 | 2 | 71.309 | 9.187 | .000 |
| Time | 211.851 | 3 | 70.617 | 9.098 | .000 |
| Colony | 27.272 | 1 | 27.272 | 3.514 | .062 |
| Treatment * Time | 74.535 | 6 | 12.423 | 1.600 | .149 |
| Treatment * Colony | 167.358 | 2 | 83.679 | 10.781 | .000 |
| Time * Colony | 20.558 | 3 | 6.853 | .883 | .451 |
| Treatment * Time * Colony | 116.826 | 6 | 19.471 | 2.509 | .023 |
| Error | 1404.867 | 181 | 7.762 | | |
| Total | 146329.253 | 205 | | | |
| Corrected Total | 2435.506 | 204 | | | |

a. R Squared = .423 (Adjusted R Squared = .350)

**Tests of Between-Subjects Effects**

Dependent Variable: RPS5

| Source | Type III Sum of Squares | df | Mean Square | F | Sig. |
|---|---|---|---|---|---|
| Corrected Model | 1627.673[a] | 23 | 70.768 | 18.040 | .000 |
| Intercept | 137922.177 | 1 | 137922.177 | 35158.452 | .000 |
| Treatment | 125.405 | 2 | 62.702 | 15.984 | .000 |
| Time | 171.905 | 3 | 57.302 | 14.607 | .000 |
| Colony | 5.390 | 1 | 5.390 | 1.374 | .243 |
| Treatment * Time | 403.570 | 6 | 67.262 | 17.146 | .000 |
| Treatment * Colony | 9.146 | 2 | 4.573 | 1.166 | .314 |
| Time * Colony | 25.766 | 3 | 8.589 | 2.189 | .091 |
| Treatment * Time * Colony | 189.486 | 6 | 31.581 | 8.050 | .000 |
| Error | 710.040 | 181 | 3.923 | | |
| Total | 173354.960 | 205 | | | |
| Corrected Total | 2337.713 | 204 | | | |

a. R Squared = .696 (Adjusted R Squared = .658)

**Tests of Between-Subjects Effects**

Dependent Variable: mGST1

| Source | Type III Sum of Squares | df | Mean Square | F | Sig. |
|---|---|---|---|---|---|
| Corrected Model | 369.771[a] | 23 | 16.077 | 7.097 | .000 |
| Intercept | 107064.971 | 1 | 107064.971 | 47261.548 | .000 |
| Treatment | 41.576 | 2 | 20.788 | 9.176 | .000 |
| Time | 68.848 | 3 | 22.949 | 10.131 | .000 |
| Colony | .055 | 1 | .055 | .024 | .876 |
| Treatment * Time | 56.428 | 6 | 9.405 | 4.151 | .001 |
| Treatment * Colony | 1.877 | 2 | .938 | .414 | .661 |
| Time * Colony | 16.516 | 3 | 5.505 | 2.430 | .067 |
| Treatment * Time * Colony | 77.335 | 6 | 12.889 | 5.690 | .000 |
| Error | 410.032 | 181 | 2.265 | | |
| Total | 138990.789 | 205 | | | |
| Corrected Total | 779.803 | 204 | | | |

a. R Squared = .474 (Adjusted R Squared = .407)

**Tests of Between-Subjects Effects**

Dependent Variable: Histone

| Source | Type III Sum of Squares | df | Mean Square | F | Sig. |
|---|---|---|---|---|---|
| Corrected Model | 411.315[a] | 23 | 17.883 | 6.434 | .000 |
| Intercept | 123964.640 | 1 | 123964.640 | 44602.419 | .000 |
| Treatment | 61.806 | 2 | 30.903 | 11.119 | .000 |
| Time | 29.579 | 3 | 9.860 | 3.548 | .016 |
| Colony | 9.291 | 1 | 9.291 | 3.343 | .069 |
| Treatment * Time | 67.512 | 6 | 11.252 | 4.048 | .001 |
| Treatment * Colony | 30.223 | 2 | 15.111 | 5.437 | .005 |
| Time * Colony | 8.913 | 3 | 2.971 | 1.069 | .364 |
| Treatment * Time * Colony | 93.853 | 6 | 15.642 | 5.628 | .000 |
| Error | 503.058 | 181 | 2.779 | | |
| Total | 167965.711 | 205 | | | |
| Corrected Total | 914.373 | 204 | | | |

a. R Squared = .450 (Adjusted R Squared = .380)
